# Molecular Dynamics Characterization of the Free and Encapsidated RNA2 of CCMV with the oxRNA Model

**DOI:** 10.1002/marc.202400639

**Published:** 2024-11-22

**Authors:** Giovanni Mattiotti, Manuel Micheloni, Lorenzo Petrolli, Lorenzo Rovigatti, Luca Tubiana, Samuela Pasquali, Raffaello Potestio

**Affiliations:** ^1^ Laboratoire Biologie Functionnelle et Adaptative, CNRS UMR 8251, Inserm ERL U1133 Université Paris Cité 35 rue Hélène Brion Paris 75013 France; ^2^ Department of Physics University of Trento via Sommarive, 14 Trento I‐38123 Italy; ^3^ INFN‐TIFPA Trento Institute for Fundamental Physics and Applications Trento 38123 Italy; ^4^ Department of Physics Sapienza University of Rome p.le A. Moro 5 Rome 00185 Italy

**Keywords:** cowpea chlorotic mottle virus, molecular dynamics, multi‐scale modeling, oxRNA model, viral RNA

## Abstract

The cowpea chlorotic mottle virus (CCMV) has emerged as a model system to assess the balance between electrostatic and topological features of single‐stranded RNA viruses, specifically in the context of the viral self‐assembly. Yet, despite its biophysical significance, little structural data on the RNA content of the CCMV virion is available. Here, the conformational dynamics of the RNA2 fragment of CCMV was assessed via coarse‐grained molecular dynamics simulations, employing the oxRNA2 force field. The behavior of RNA2 was characterized both as a freely‐folding molecule and within a mean‐field depiction of the capsid. For the former, the role of the salt concentration, the temperature and of ad hoc constraints on the RNA termini was verified on the equilibrium properties of RNA2. For the latter, a multi‐scale approach was employed to derive a potential profile of the viral cavity from atomistic structures of the CCMV capsid in solution. The conformational ensembles of the encapsidated RNA2 were significantly altered with respect to the freely‐folding counterparts, as shown by the emergence of long‐range motifs and pseudoknots. Finally, the role of the N‐terminal tails of the CCMV subunits is highlighted as a critical feature in the construction of a proper electrostatic model of the CCMV capsid.

## Graphical abstract

The cowpea chlorotic mottle virus (CCMV) is an exemplary and well‐studied ssRNA virus, yet as of now little structural data on its RNA content is available. Here, coarse‐grained molecular dynamics simulations and mean‐field modeling strategies are employed to shed light on the conformational dynamics of the RNA2 fragment of CCMV and the interplay between its electrostatic and topological features.

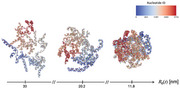

## 1 Introduction

Viruses are nano‐sized pathogenic “organisms”, capable of self‐replicating by hijacking the molecular machinery of a host cell—this process affecting humans, livestock, plants, and bacteria alike.^[^ ^1^ ^]^ Yet, they constitute a potential technology for pharmaceutical applications, e.g., as vectors for vaccines:^[^ ^2^ ^]^ In all respects, viruses are natural nano‐machines whose activity has been fine‐tuned by evolution, thereby representing a model system to benchmark fundamental biophysical theories on RNA‐protein interactions, protein–protein interactions, and on the interplay between evolution and the physico–chemical constraints enforced on simple genomes.^[^ ^3^, ^4^ ^]^

The simplest viruses—such as the brome mosaic virus (BMV), the cowpea chlorotic mottle virus (CCMV), and the MS2 bacteriophage – are composed of a handful of proteins describing an icosahedral shell, or capsid, about a long single‐stranded RNA (ssRNA) molecule. It has been shown that solutions of viral RNAs and capsid proteins might spontaneously self‐assemble into mature, infective viruses in vitro, at proper pH and salt concentration.^[^ ^4^, ^5^, ^6^, ^7^ ^]^ Furthermore, when different RNA molecules are involved, e.g., in a competitive scenario, most viruses are capable to selectively package their homologous RNA in an extraordinarily efficient manner.^[^ ^3^, ^4^, ^8^ ^]^

The self‐assembly of single‐stranded RNA viruses has been the object of intense experimental, theoretical, and computational research over the past fifteen years.^[^ ^3^, ^4^, ^8^, ^9^, ^10^, ^11^, ^12^, ^13^, ^14^ ^]^ While diverse aspects are still open for debate, a general picture has emerged, whereby the viral assembly follows a nucleation‐and‐growth process, mostly driven by non‐specific electrostatic forces: Yet, there are evidences^[^ ^15^ ^]^ that this process is sometime aided by selective, short‐range interactions taking place between highly‐conserved regions of RNA, or packaging signals (PSs), and the capsid proteins (CPs). These interactions are critical in both starting the self‐assembly and encapsidating the correct (homologous) RNA. For example, the co‐assembly between capsid subunits and the unique PS in the RNA of the helical Tobacco Mosaic Virus (TMV) yields a nucleation seed,^[^ ^16^ ^]^ which further grows into a mature capsid most likely via non‐specific electrostatic interactions. A more complex scenario unfolds in MS2, with several PSs attaching onto specific sites of the capsid proteins;^[^ ^17^, ^18^, ^19^ ^]^ these are likely related to the precise organization of RNA in the capsid, which allowed the cryo‐EM imaging of more than 90% of the viral RNA content of MS2.^[^ ^20^ ^]^ In other viruses, the existence of PSs has been only hinted (e.g., in BMV), or none have been found altogether—which is the case of CCMV – thereby suggesting that the self‐assembly process is dominated by non‐specific, electrostatic forces between RNA and the CPs.^[^ ^3^, ^4^ ^]^

As shown by experimental and theoretical works, specifically on CCMV, viruses are nevertheless expected to discriminate between RNA substrates upon their shape and diameter, even in the absence of selective interactions:^[^ ^8^, ^10^ ^]^ This capability is accounted for by both the degree of branching of the RNA molecules^[^ ^11^, ^12^, ^13^ ^]^ and the interactions between RNA and a set of disordered, positively‐charged arginine‐rich motifs (ARMs), lining the inner walls of diverse viral capsids.^[^ ^21^, ^22^ ^]^ In fact, it appears that viral RNAs have been subject to evolutionary pressures affecting not only their sequence and conserved folded motifs,^[^ ^17^, ^18^, ^23^, ^24^, ^25^ ^]^ but their size, shape, and branching degree alike.^[^ ^26^, ^27^, ^28^, ^29^, ^30^, ^31^ ^]^

CCMV is composed of an icosahedrally‐symmetric capsid of 180 identical CPs, showing a radius of about 28 nm and a triangulation number T = 3, according to the Caspar–Klug classification.^[^ ^32^ ^]^ Its genome is tripartite, i.e., a native CCMV capsid either packages a single copy of RNA1 (about 3000 nt long), RNA2 (about 2800nt), or co‐packages a copy of RNA3 (2100nt) and RNA4 (700nt).^[^ ^33^ ^]^ Notably, CCMV spontaneously assembles about a variety of polyelectrolytes, such as polystyrene sulfonate, poly‐uracyl chains, and other heterologous viral RNAs, making it a model virus to characterize the role of non‐specific interactions in the assembly process. In fact, this broad adaptability has driven experimentalists to perform competition assays, whereby different RNA moieties compete for a limited amount of CCMV CPs.^[^ ^8^, ^33^ ^]^ Somewhat surprisingly, it was shown that the RNAs of CCMV are outcompeted by heterologous RNA molecules of similar length belonging to, e.g., BMV.^[^ ^8^ ^]^ Moreover, the best cargo of the CCMV vessel appears to be a fully linear polyelectrolyte, although the latter yields smaller capsids, compatible with a triangulation number *T* = 2.^[^ ^33^ ^]^ Lastly, as with many other viruses, CCMV is overcharged,^[^ ^34^ ^]^ i.e., its RNA bears a significantly higher electrical charge than that of its capsid.^[^ ^35^ ^]^ This fact has been explained in the light of the interaction between the highly positively charged ARMs and RNA.^[^ ^21^ ^]^

Despite their biophysical significance, the only data on the properties of encapsidated CCMV RNA at our disposal come from either theoretical assessments based on mean‐field descriptions of the cargo^[^ ^11^, ^12^, ^13^ ^]^ or from coarse‐grained (CG) simulations, where the RNA is mapped to (branched) chains of beads.^[^ ^21^ ^]^ In other viruses, such as MS2 and STMV, the exact position of a large portion of the genome is known from experiments,^[^ ^20^, ^36^, ^37^ ^]^ thereby enabling for refined and rigorous simulation approaches^[^ ^38^, ^39^ ^]^; this protocol, however, has not been applicable to CCMV, where no structural, experimental data on RNA is available.

With the recent development of coarse‐grained models of RNA, such as oxRNA, and the computational speed‐up brought by GPU computing, one might now assess the dynamical evolution and the (structural, topological) ensemble properties of viral RNAs at an adequate level of resolution,^[^ ^40^, ^41^, ^42^ ^]^ as an alternative to conventional approaches of secondary structure prediction, such as RNAfold of the ViennaRNA package.^[^ ^43^ ^]^

In this work, we embrace this approach and employ coarse‐grained simulations to explore the conformational dynamics of the single‐stranded RNA2 fragment of CCMV as a freely folding molecule in solution and within a capsid‐like enclosing sphere. For the former, we verify how the equilibrium, conformational properties of the RNA2 are affected by the salt concentration, the temperature, and the application of an harmonic constraint that forces the 3' and 5' termini within a few nanometers from one another. In fact, the latter is motivated by earlier experimental and theoretical works inferring that the ends of long RNAs shall be spontaneously close.^[^ ^44^, ^45^ ^]^

For the latter, we deploy two mean‐field depictions of the viral capsid: A first approach relies on an analytical solution of the Poisson–Boltzmann equation for an empty icosahedral shell, whereas in a second approach we use Gauss' theorem to map the charge distribution of the atomistic structure of the CCMV capsid (and the ionic shells thereof) into a spherically‐symmetric, radial potential profile. By these means, we assess a decisive role of the electrostatic forces—from both the saline medium and the capsid—that reflect in a diverse conformational dynamics and stability of the RNA2 molecules. In spite of a mild confinement process, the encapsidation enforces a significant bias on the structural ensemble of RNA2, enabling stable long‐range interactions (such as pseudo‐knots) that are not observed in a freely folding scenario. Lastly, we discuss the capabilities and limitations of adopting effective and mean‐field approaches, and of the numerical tools at our disposal.

## 2 Experimental Section

### 2.1 The oxRNA2 Force Field

oxRNA2 is a coarse‐grained force field, first developed for applications to RNA nanotechnology, and derived via a top‐down approach based on the structural, mechanical, and thermodynamic properties of RNA.^[^ ^42^, ^46^ ^]^ Each nucleotide is associated with a single rigid body and two interaction sites, which take into account hydrogen‐bonding and stacking forces between CG sites, the backbone connectivity, and excluded volume interactions.

The oxRNA2 force field effectively models the major and minor grooving of the RNA helices, as well as a salt‐dependent modulation of the electrostatic interactions via a Debye–Hückel formulation, thereby adapting the dynamics of RNA in implicit solvent to diverse concentrations of monovalent salt.^[^ ^42^ ^]^ Moreover, it offers a sequence‐dependent description of the strength of stacking and (Watson–Crick, wobble) hydrogen‐bonding interactions, fitted upon the melting temperature of RNA duplexes and based on the nearest‐neighbour models of SantaLucia and Turner.^[^ ^47^, ^48^ ^]^

Last, despite lacking a proper definition of non‐canonical interactions—such as Hoogsteen and sugar‐edge hydrogen bonds, and ribose zippers—oxRNA2 consistently displays a variety of tertiary motifs, namely the coaxial stacking of RNA helices, kissing‐loops, and pseudo‐knots.^[^ ^40^ ^]^

### 2.2 Molecular Dynamics Simulation Protocol

As mentioned in the **Introduction** section, CCMV is a multi‐partite ssRNA virus, i.e., its genome is packed within three separate capsid vessels, all of which are required by the infective process: Out of four CCMV viral fragments, we chose RNA2, whose gyration radius in solution has been estimated by scattering techniques;^[^ ^49^ ^]^ The 2774‐nt sequence of this fragment is detailed in Section I (Supporting Information). All molecular dynamics (MD) simulations of the RNA2 molecule were performed employing the oxRNA2 force field, at diverse concentrations of monovalent salt—namely 0.15 and 0.5 M—within the single‐GPU, native implementation of the oxDNA simulation code.^[^ ^41^, ^50^ ^]^

The adopted MD protocol is subdivided into three stages: 1) a first equilibration and relaxation of the RNA2 molecule from a linear conformation; 2) a confinement stage, which we here dub as *squeezing*, whereby we steadily enclose the RNA2 molecule within a spherical volume that is compliant with the size of the inner cavity of the CCMV capsid; 3) the dynamics of the encapsidated RNA2 within a mean‐field depiction of the electrostatic field of the CCMV capsid and the ionic shells thereof. Hence, ad hoc simulation protocols were designed as follows, according to the specific requirements of each stage.

#### Structural Equilibration of the RNA2 molecule

The CG coordinates of a linear conformation of the single‐stranded RNA2 molecule were obtained by the TacoxDNA software^[^ ^51^ ^]^; Thus, a twofold equilibration procedure was applied.

Firstly, we performed a swift relaxation in the NVT ensemble via the Bussi thermostat,^[^ ^52^ ^]^ thereby obtaining decorrelated conformations of the RNA2 molecule at both salt concentrations (that is, 0.15 and 0.5 M). As for this stage, MD simulations of 2 × 10^8^ steps were carried out at the constant temperature of *T* = 310 K, employing a timestep δ*t* = 3 × 10^−3^ τ ≃ 9 fs—with τ defining the internal simulation time unit. The correlation time and timestep frequency of the Bussi thermostat were set to 1000 and 53 respectively—as suggested by the software developers.

In addition, hydrogen‐bonding interactions were switched off, to try and avoid that the RNA2 conformations were significantly biased by the relaxation pathway in the subsequent MD stages. We thus tracked the gyration radius (*R*_*g*_) along the trajectories and extracted three, non‐correlated RNA conformations per salt concentration, about the minimum (I), average (II), and maximum (III) values of *R*_*g*_(*t*) in the late plateau of the Bussi dynamics.

Second, we performed a Langevin MD simulation of each (six) selected RNA2 conformation (*T* = 310 K, 1 × 10^10^ steps), hereby referred to as *freely‐folding* MD: Values of the diffusion coefficient and timestep frequency associated with the Langevin thermostat were set to 2.5 and 103 respectively, while the MD timestep δ*t* = 3 × 10^−3^ τ was kept. At this stage, hydrogen‐bonding and sequence‐specific interactions were switched back on, thereby letting the systems fold into secondary and tertiary structural motifs. In fact, no secondary structure has ever been imposed on the system a priori.

A similar protocol was followed for the setup of the freely folding MD scenarios with harmonically bound RNA termini (see the discussion in the **Results and Discussion** section)—but for a few choices: A circular RNA arrangement was adopted as starting conformation of the Bussi dynamics, whereby the terminal nucleotides are driven smoothly apart (to a reference distance of 5λ_*ox*_ ≈ 4 nanometers) via a harmonic force associated with $k=0.5\;k_{b}T\lambda_{ox}^{-2}$. This bias is enforced throughout the equilibration stage (Bussi) and freely folding (Langevin) dynamics, and kept upon the squeezing/encapsidation protocol.

#### Squeezing of the RNA2 Molecule by a Spherical Confinement

To mimic a CCMV environment, the RNA2 molecule was first confined within a spherical volume that is compliant with the size of the inner cavity of the CCMV capsid. To this aim, we employed a radially‐symmetric, time‐dependent harmonic potential, acting effectively upon the nucleotides that lie about the enclosing surface, and defined as:

(1)

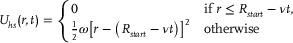

where *r* is the radial coordinate within a reference frame that is centered at the center of mass of the model capsid, $\omega =1\;k_{B}T\lambda_{ox}^{-2}$ defines the stiffness of the enclosing sphere (with *T* = 310*K* and the “*ox*” subscript denoting the internal units employed by oxRNA2 — refer to the Supporting Information of ref. [40] for details), and ν and *R*_*start*_ are the encapsidation rate and starting radius associated with the squeezing regime respectively. All MD replicas were set up to achieve a final encapsidation radius between 10 and 12 nm.

MD simulations were performed in the NVT ensemble at 310 K via a Langevin thermostat: The diffusion coefficient and timestep frequency of the thermostat, and the simulation timestep of the freely‐folding setup were kept. For the sake of the numerical efficiency, this squeezing protocol has been applied to those RNA conformations showing the lowest value of the gyration radius during the freely folding stage within each (I, II, III) MD replica per salt concentration.

For the enclosing potential to be the least disruptive of the molecular structure of the RNA2, we set a value of ν = 5.6 × 10^−8^ λ_*ox*_(MDsteps)^−1^, while making sure that the internal pressure of the system did not diverge throughout the simulations (see Figure S15, Supporting Information). As for the 0.5 M scenario, a satisfactory value of the encapsidation radius was achieved, exerting no excess external force on the RNA structure. In fact, this rate was adopted in the setup of the MD scenarios with harmonically bound RNA termini alike.

**Figure 1:**
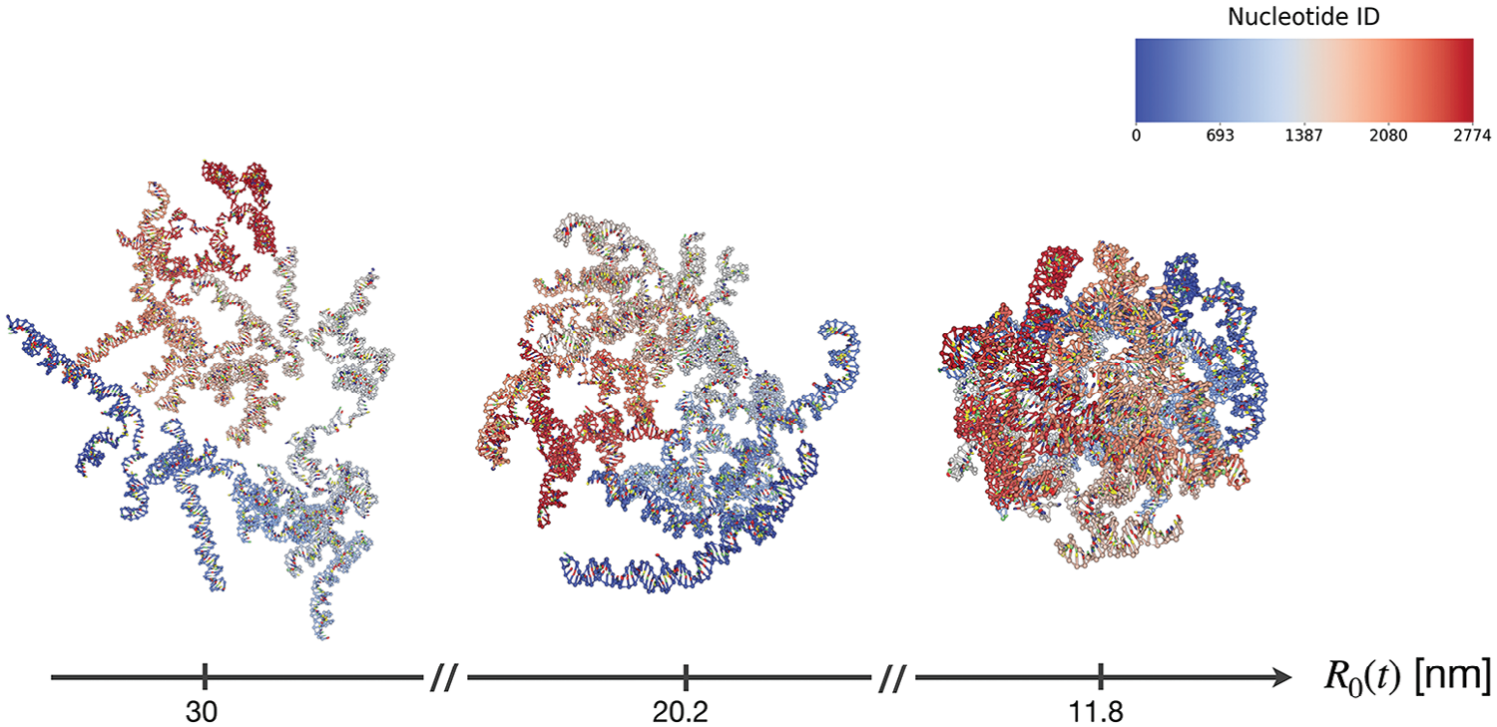
Snapshots of an example *squeezing* stage for an RNA2 molecule at 0.5 M—values of the instantaneous radius of the enclosing sphere are reported below. The color scale is associated with the nucleotide index.

Yet, to tolerate the squeezing stage, the 0.15 M RNA structures required a slightly milder procedure, i.e., with ν reset to 1 × 10^−8^ λ_*ox*_(MDsteps)^−1^ about halfway of the MD trajectory. **Figure** **1** exemplifies the squeezing stage associated with RNA2 at 0.5 M.

#### 2.2.1 Dynamics of the Encapsidated RNA2 Within a Mean‐Field External Potential

The sole squeezing procedure yields an ensemble of confined, strained RNA2 conformations, which shall subsequently adjust to the electrostatic field of the CCMV capsid. To this concern, we adopted two mean‐field approaches, based on a static depiction of the capsid and the solvent, to mimic the inner environment of the CCMV cavity. A first approach, denoted *U*_*yuk* + *WCA*_(*r*), is based on the analytical formulation reported by Šiber and Podgornik^[^ ^53^ ^]^ and subsequently adopted in other theoretical studies:^[^ ^54^ ^]^ The Poisson–Boltzmann equation is solved for the electrostatic potential associated with an empty icosahedral capsid, depicted as a spherical thin shell, at a fixed monovalent salt concentration. By employing the Debye–Hückel approximation, an analytical solution is derived that applies to both the internal and the external environment of the viral capsid.

**Figure 2:**
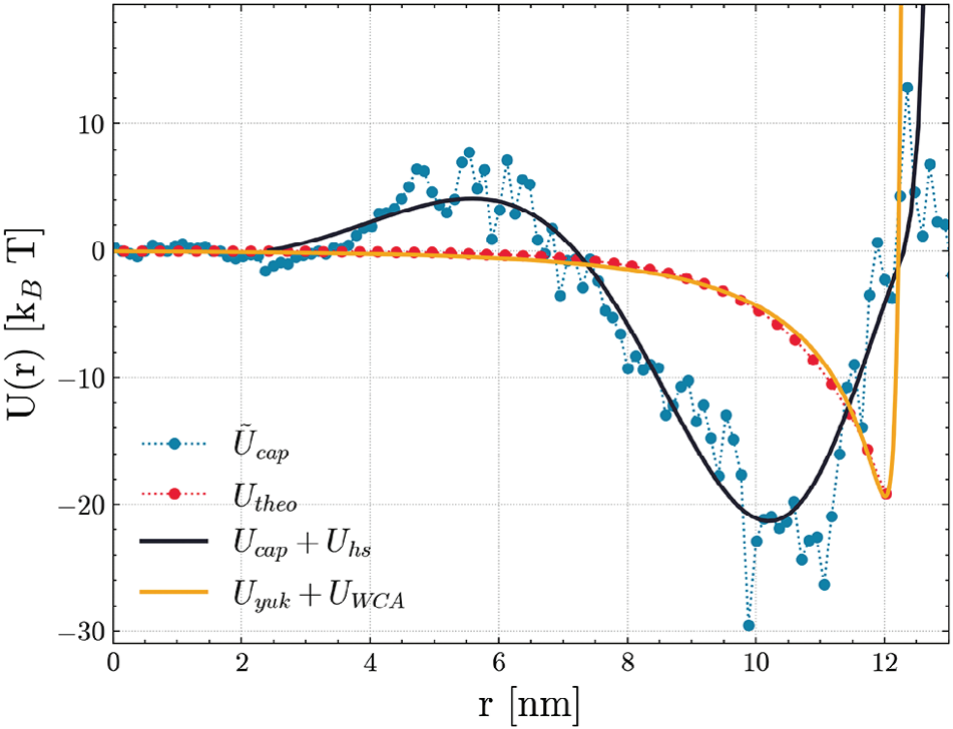
Radial potential energy profiles of the CCMV capsid and the ionic shells thereof, which have been adopted for the dynamics of the encapsidated RNA2 (depicted as solid lines). Data points are associated with the analytical (red dots) and the structure‐based approach (blue dots) respectively.

Here, we fitted this solution via a combination of a Yukawa attractive potential and a short‐range WCA repulsive wall (see **Figure** **2**), as:

(2) $$\begin{array}{ccc} & & U_{yuk+WCA}\left(r\right)=U_{yuk}\left(r\right)+U_{WCA}\left(r\right)=\frac{\alpha \exp\left[-(R_{\delta }-r)/\lambda \right]}{\left(R_{\delta }-r\right)}\\ & & +4\varepsilon \left[{\left(\frac{\sigma }{\left(R_{\delta }-r\right)}\right)}^{12}-{\left(\frac{\sigma }{\left(R_{\delta }-r\right)}\right)}^{6}\right]+\varepsilon \end{array}$$

with *R*_δ_ (a slightly higher value of) the radius of the CCMV cavity; α and λ the amplitude and Debye length of the Yukawa potential; ϵ and σ the amplitude and radius of the standard WCA potential. Parameters of the fitting procedure were implemented within the oxDNA simulation code and are reported in Section IV.D (Supporting Information).

**Figure 3:**
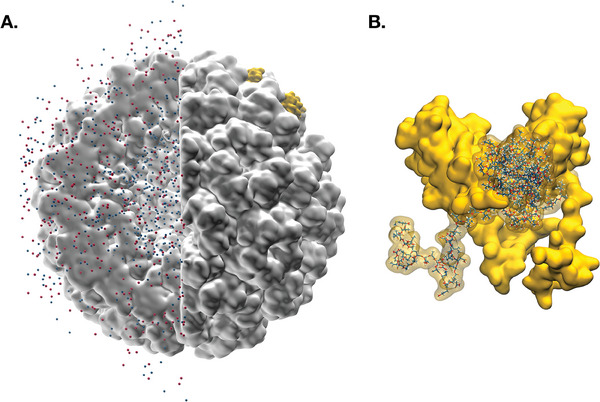
A) Snapshot of the CCMV capsid and the thermalized shells of sodium (red) and chloride (blue) ions thereof. B) Surface depiction of the trimeric symmetrical subunit of CCMV, along with the amino‐terminal tails decorating the inner capsid walls.

The second approach, denoted *U*_*cap*_(*r*), was based on a multiscale protocol developed specifically for this work, whereby we employed the full structure of the CCMV capsid at the atomistic level of resolution to extract a mean‐field, radial potential profile (details are discussed in Section IV.B, Supporting Information). Briefly:

- upon the Protein Data Bank template (entry ID: 1CWP, X‐ray crystallographic structure obtained at pH 5^[^ ^55^ ^]^), the amino‐terminal tails of the trimeric CCMV capsomer (i.e., the elementary symmetrical unit of the icosahedral capsid shell) were built via the Modeller plugin^[^ ^56^ ^]^ of the Chimera software,^[^ ^57^ ^]^ and equilibrated both in vacuum and in solution (see **Figure** **3**B);
- the newly‐achieved trimer subunit was replicated according to the sixty‐fold symmetry of the icosahedral shell, yielding an all‐atom structure of the CCMV capsid;
- the latter was subject to diverse rounds of energy minimization—both in vacuum and in solution, hence the solvent medium was thermalized via a multi‐step MD protocol. Figure 3 shows a snapshot of the final conformation of the system.

We thus defined a radial potential energy profile upon the partial charge distribution of both the viral capsid and the thermalized ionic shells, corresponding to the last configuration obtained by the above‐described protocol. To this purpose, we iteratively employed Gauss' theorem, relying on the approximation that the system was spherically symmetric about the center of mass of the capsid. The charge distribution has been averaged over the solid angle and discretized along the radial coordinate, thereby achieving the values of ${\tilde{U}}_{cap}\left[r\right]$ shown in Figure 2.

Lastly, we fitted ${\tilde{U}}_{cap}\left[r\right]$ against an infinitely differentiable analytic function *U*_*cap*_(*r*) of the form:

(3) $$U_{cap}\left(r\right)=e^{A(R_{0}-r)}\cdot \left(\sum_{n=1}^{N}c_{n}\cdot {\left(R_{0}-r\right)}^{n}\right)$$

with *N* = 10 and *R*_0_ the radius of the inner CCMV cavity (likewise, parameters of the fit are reported in Section IV.D, Supporting Information).We note here that the structure of the CCMV capsid employed is stable at pH ∼ 5, whereas the charges of the residues on the capsid tails have been assigned within the assumption of physiological (neutral) pH. This notwithstanding, their value—mostly associated with basic amino acids—is not expected to vary between pH 4 and 8.^[^ ^58^ ^]^ Finally, we included a further harmonic potential about the capsid walls, as in Equation (1) but with no time‐dependence and *R*_*start*_ = *R*_0_: This term mimics an excluded volume interaction, hence preventing the nucleotides from overcoming the fictitious capsid shell. Results of the fitting procedure are shown in Figure 2.

### 2.3 Analysis Protocol

Analyses have focused on diverse structural features of the RNA2 configurational ensembles. Values of the hydrogen‐bonding interactions, potential energy, and internal pressure of the RNA2 structure were calculated with the oxDNA software tools. Contact maps were built on a frame‐wise basis as Boolean matrices of the base pairings between nucleotides, whereby we tracked the evolution of the configurational ensembles of RNA2 and identified stable contacts, i.e., hydrogen‐bonding interactions that are conserved over 50% of the (total, individual) MD trajectories.

A distance criterion based on the Hamming distance between the upper‐triangular portion of two contact maps (i.e., counting the number of different entries) was adopted as metric of a hierarchical clustering—together with an average linkage criterion. Lastly, we relied on in‐house scripts for all calculations and figures, based on oxDNA analysis tools^[^ ^59^ ^]^ and (scientific) Python libraries.^[^ ^60^, ^61^, ^62^, ^63^, ^64^, ^65^, ^66^ ^]^

## 3 Results and Discussion

### 3.1 Freely Folding Dynamics of the RNA2 Fragment of CCMV in Solution

**Figure 4:**
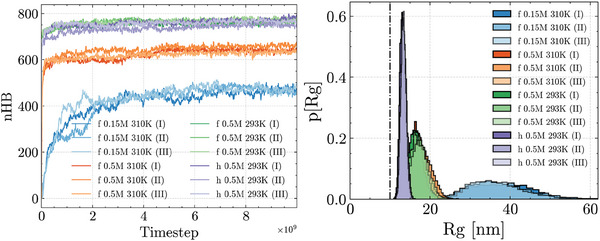
(Left) Evolution of the total amount of hydrogen‐bonding interactions (nHB) and of (Right) the gyration radii of the RNA2 molecules, from the freely‐folding MD at 0.15 and 0.5 M (all scenarios): Higher (effective) salt concentrations and lower temperatures account for an increase in the nucleotide pairings, while fixing a harmonic restraint between the RNA termini yields a further decrease in the gyration radii of RNA2 (“h” curves). We note that all MD replicas per salt concentration converge to a consistent (nHB, *R*_*g*_) plateau, regardless of the starting structure. The dot‐dashed line marks the estimate value of the gyration radius of RNA2 reported in ref. [49]. The labels “f” and “h” refer to the disjointed (free) and restrained (harmonic) RNA termini scenarios respectively.

First, we characterized the behavior of a freely folding RNA2 molecule in solution, at 310 K and diverse monovalent salt concentrations—i.e., 0.15 and 0.5 M—this being one tunable feature of the oxRNA2 force field. Although experimental works have shown that the CCMV does not assemble at 0.5 M in vitro (see e.g., ref. [67]), it is known that the local saline concentration about the backbone of RNA molecules might be higher than in the bulk medium.^[^ ^68^, ^69^ ^]^ We thus employ a 0.5 M setup as an effective way to account for the enhanced electrostatic shielding from the ionic cloud: In fact, this choice is strictly necessary in coarse‐grained models such as oxRNA2, where the electrostatic interactions are modeled as a Debye–Hückel potential with implicit counterions. As described in the **Experimental** Section, the RNA2 molecule has been subject to an equilibration protocol that allowed us to achieve an ensemble of non‐correlated RNA configurations: This way, we would setup the freely‐folding MD out of a diversified pool of starting structures, thus avoiding that the RNA2 molecules be stuck into locally‐folded states. As the hydrogen‐bonding interactions are switched back on, the pairing of nucleotides drives a broad stabilization of the internal energy, together with a decrease in the gyration radius of the RNA molecules (as shown in **Figure** **4**).

Overall, the 0.15 and 0.5 M scenarios display diverse conformational dynamics—the latter showing a higher amount of hydrogen‐bonding interactions and degree of compactness. This is in line with the Debye–Hückel interactions screening the electrostatic repulsion less effectively at a lower salt concentration. Yet, it is significant that all MD replicas per scenario converge to a consistent value of number of contacts (about 475 hydrogen bonds at 0.15 M versus 650 hydrogen bonds at 0.5 M) and gyration radius (about 35 nm at 0.15 M vs 17.5 nm at 0.5 M), regardless of the starting structure.

Notably we observe a discrepancy between the values of the gyration radii of RNA2 from the freely folding MD at 310 K and the estimates of *R*_*g*_ ∼ 10 nm reported by Marichal and co‐workers from small‐angle X‐ray scattering measurements in solution^[^ ^49^ ^]^: This outcome is partly accounted for by the different state point adopted in the experimental setup, with *T*_*exp*_ = 293 K and a nominal monovalent salt concentration of about 0.1 M (see Section 3.3). Likewise, this discrepancy might be associated with the simulation protocol adopted—although a trajectory based on a simulated annealing procedure yielded similar values of the gyration radii (see Figure S12, Supporting Information). Last, we verified the structural role of the application of a harmonic constraint forcing the 3' and 5' termini within a few nanometers from one another—i.e., an experimental evidence^[^ ^44^ ^]^ that is not independently recovered by oxRNA2. The results (curves “h” in Figure 4) will be described in detail in Section 3.3.

**Figure 5:**
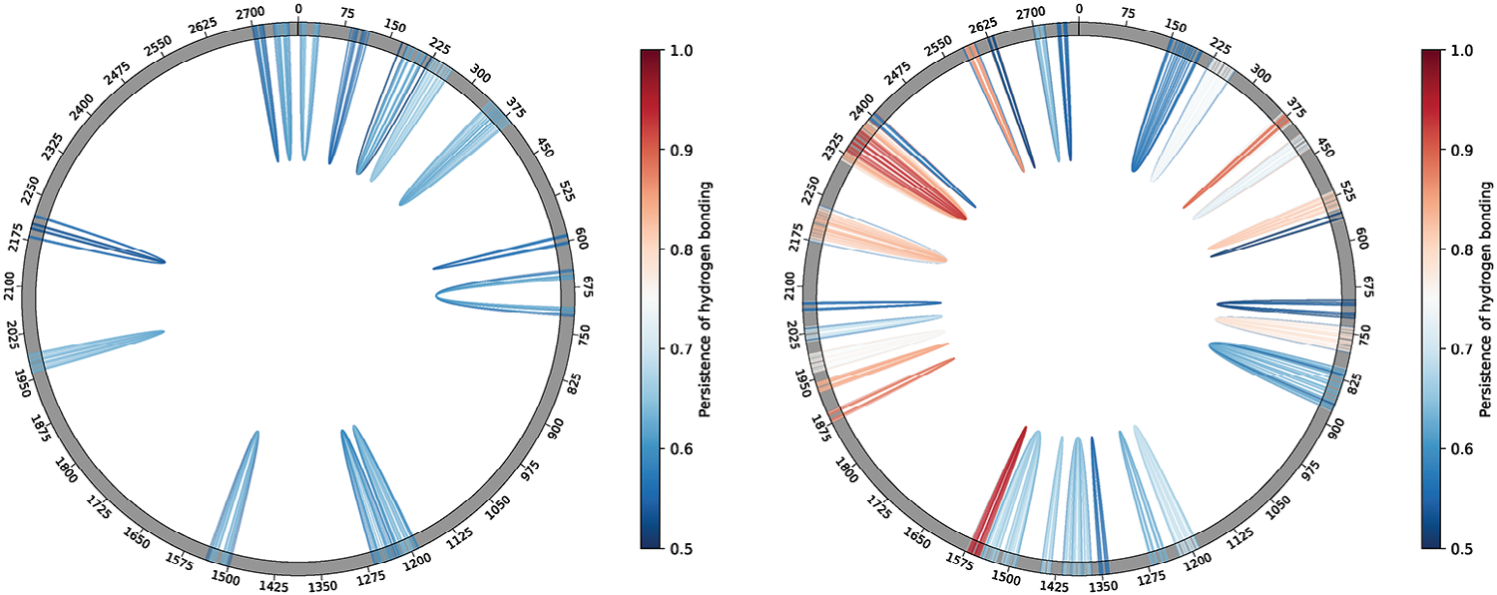
Chord diagram depiction of the stable hydrogen‐bonding contacts (i.e., conserved in over 50% of the total trajectory frames per salt concentration), from the equilibrated fraction of the freely‐folding MD at 310 K and (left) 0.5 M or (right) 0.15 M.

In addition, Figure 4 shows that the quality and kinetics of the folding process are strongly salt‐dependent. In fact, the trends observed at 0.5 M highlight a steep rise in the amount of hydrogen‐bonding interactions and a subsequent decrease in both the internal energy and the gyration radius of the RNA molecules, consistently leading to deep and narrow free energy basins (see Figure S7, Supporting Information). This is associated with a high variability in the stable contacts, which are diversely distributed among MD replicas ‐ as shown by the chord diagrams in both **Figure** **5** and Figure S8 (Supporting Information). We thus observe that the free RNA molecule achieves a well‐defined thermodynamic state at high ionic strength, characterized by narrowly distributed values of global observables—such as the energy and gyration radii—which nonetheless emerge from a pool of diverse conformations at the molecular level.

Conversely, at 0.15 M a slower, steadier increase in the amount of hydrogen‐bonding interactions accounts for broader and highly degenerate free energy landscapes, whereby the RNA molecules have access to a range of states lying within ΔR_*g*_ = 10 − 15 nm and ΔU = 0.3 − 0.5 k_*B*_T. However, a non‐negligible fraction of the stable contacts scored at 0.15 M are shared between all MD replicas, as if all folding pathways somewhat consistently converged to an array of locally folded secondary structures (see Figure 5; Figure S8, Supporting Information).

At the level of single MD replicas, hydrogen‐bonding interactions between nucleotides span a wider range at 0.5 M than at 0.15 M, with the distribution of distances shifting towards higher values in the former case. However, we note that, under a freely folding regime at 310K and at both salt concentrations, no stable long‐range interactions nor pseudoknots are observed—whose occurrence is expected both in functional (such as rRNAs and tRNAs^[^ ^70^ ^]^) and in viral RNA fragments.^[^ ^71^, ^72^ ^]^

These observations are corroborated by the analysis of the contact maps between nucleotides, which display a dynamical evolution underneath a seemingly static equilibrium: In fact, at both salt concentrations, the systems progresses via a steady redistribution of the hydrogen‐bonding interactions, as shown by both Figure S6 (Supporting Information) and the distance matrices in Section II.B (Supporting Information). These matrices, together with the hierarchical clustering of the contact maps (see the dendrograms in Figures S4 and S5, Supporting Information), recapitulate our earlier observations on the non‐trivial salt dependency of the conformational landscapes of RNA2: despite both scenarios exploring diverse structural ensembles (with no apparent overlap between secondary/tertiary motifs amongst MD replicas), the distances between contact maps at 0.5 M are about twice as high as they are at 0.15 M.

### 3.2 Dynamics of RNA2 Within a Capsid‐Like Electrostatic Environment

We thus tracked the dynamics of RNA2 at 310 K within the constraints enforced by a capsid‐like environment. As detailed in the Experimental Section, the encapsidation of the RNA2 molecules involves two stages: i) the radial confinement of RNA2 (squeezing), to achieve molecular structures that are compliant with the size of the CCMV cavity – associated with an inner radius *R*_0_ = 12 nm, and ii) its adjustment to the external field of the CCMV capsid.

The external harmonic potential of the squeezing stage was fixed as a convenient trade‐off between a quasi‐static scenario—that is, the least disruptive of the molecular topology—and the numerical efficiency of the calculation (refer to Section 2.2 Supporting Information). Yet, we observed that the spatial proximity enforced by the squeezing procedure drives a significant rise in the amount of hydrogen‐bonding interactions: This effect is steadier in the 0.5 M scenario and abrupt at 0.15 M, yielding a 60% contact increase in the latter case (as shown in Figure S16, Supporting Information).

**Figure 6:**
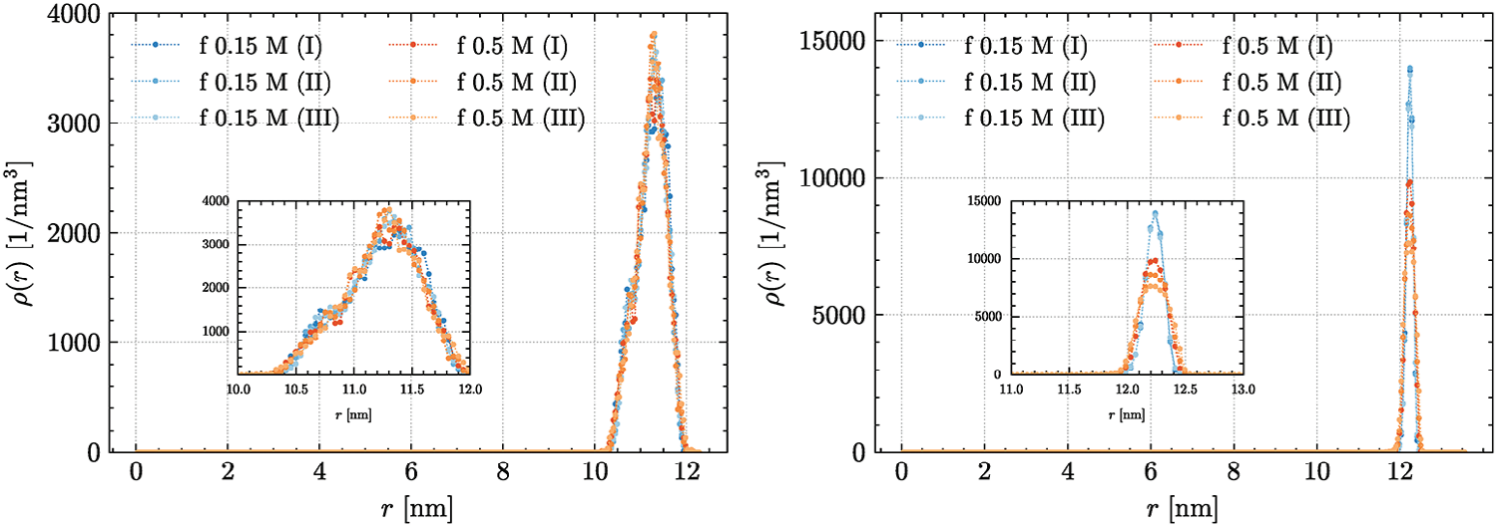
Profiles of the radial nucleotide density for the MD trajectories of RNA2 at 310 K within a capsid‐like electrostatic environment, derived via mean‐field approaches relying on either (**left**) atomistic data (structure‐based approach), or (**right**) theoretical calculations (analytical approach)—details in the main text.

Therefore, two mean‐field approaches were adopted to depict the electrostatic environment of the inner CCMV cavity, denoted as *U*_*yuk*_(*r*) and *U*_*cap*_(*r*) (Equations 2 and 3 in Section 2.2.1, Experimental Section, respectively). In both scenarios, and at both salt concentrations, the RNA2 molecules adjust to the external field within about 1 × 10^8^ MD steps, thereby (virtually) adhering to the internal walls of the CCMV capsid. Yet, the radial distribution of nucleotides (**Figure** **6**) is broader in the *U*_*cap*_(*r*) scenario, whereas *U*_*yuk*_(*r*) confines RNA2 to a thin layer about the inner capsid walls. We note that the former is qualitatively in line with both the theoretical calculations reported by Marichal and co‐workers^[^ ^49^ ^]^ and with the data of Zhang and co‐workers,^[^ ^73^ ^]^ thereby highlighting the critical role of the N‐terminal tails in the description of the electrostatic environment—even only by a mean‐field approach.

**Figure 7:**
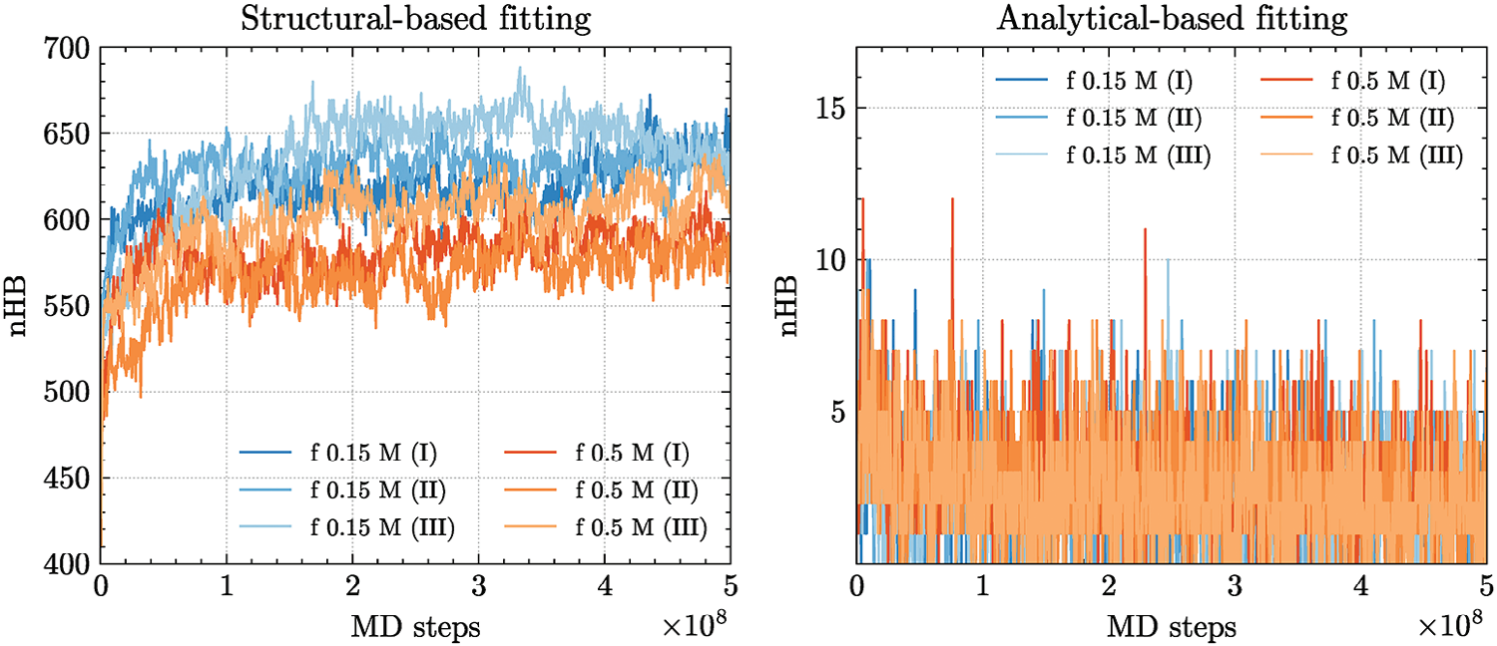
The total amount of hydrogen‐bonding interactions (nHB) from the MD trajectories of RNA2 at 310 K within a capsid‐like electrostatic environment, obtained by applying either (left) a structure‐based (*U*_*cap*_(*r*)) or (right) an analytical approach (*U*_*yuk*_(*r*)) (details in the text).

In fact, the *U*_*yuk*_(*r*) external potential enforces a systematic disruption of the nucleotide pairings and RNA secondary structures (see **Figure** **7**)—which tallies, however, with the discussion in ref. [12].

**Figure 8:**
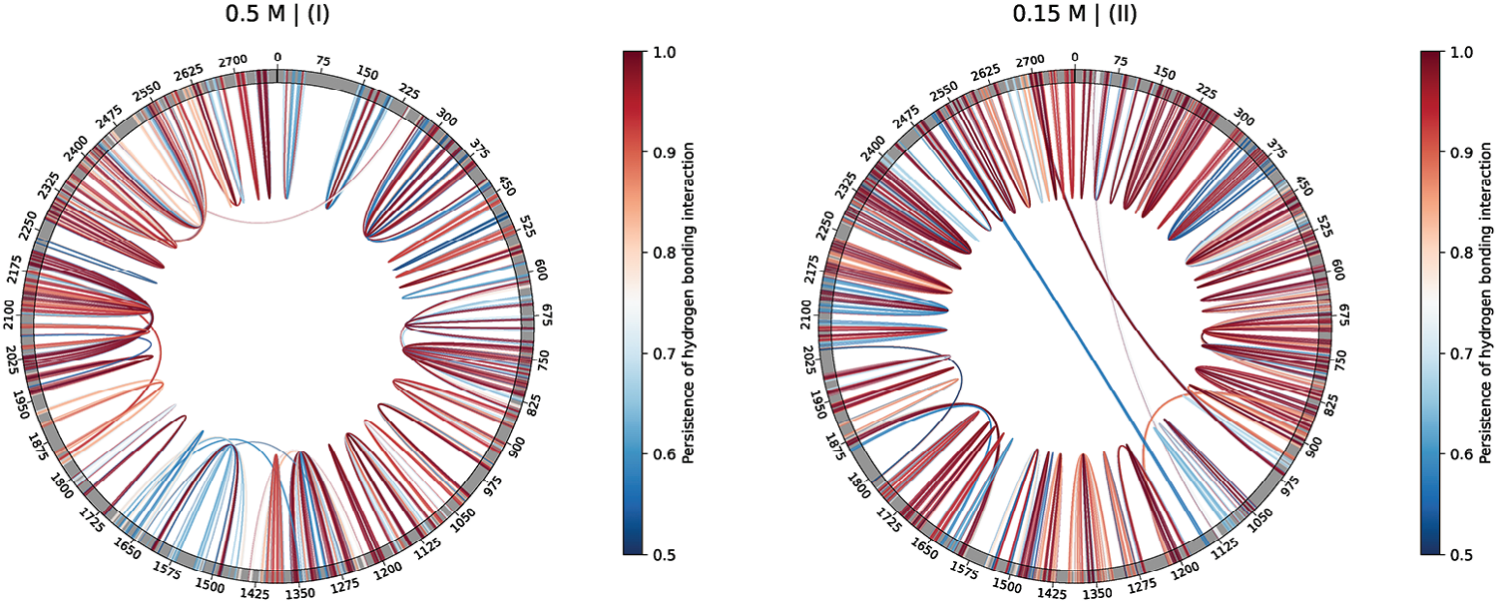
Chord diagram depiction of the stable hydrogen‐bonding contacts, i.e., conserved in over 50% of the trajectory frames, corresponding to (left) replica I at 0.5 M (310 K), and (right) replica II at 0.15 M (310 K), within the *U*_*cap*_(*r*) capsid‐like, electrostatic environment (details in the main text).

Conversely, not only is this behavior not recapitulated by applying *U*_*cap*_(*r*), but the amount of hydrogen‐bonding interactions builds up in the latter case, as shown by Figure 7. Moreover, within the external field of the *U*_*cap*_(*r*) CCMV capsid, the 0.15 M scenarios slightly outrank the 0.5 M counterparts over the total amount of nucleotide pairings, thereby suggesting that the CCMV environment might outcompete the electrostatic stabilization of the saline medium. The structural enhancement driven by *U*_*cap*_(*r*) yields a rich variety of contacts and long‐range motifs, such as RNA pseudoknots, in both the 0.15 and 0.5 M scenarios (as shown by **Figure** **8**). As observed earlier Section 3.1), several stable contacts are systematically found in all MD replicas at 0.15 M (see Figure S21, Supporting Information). Notably, a significant fraction of these contacts is identically found in the freely folding trajectories: Thus, despite the squeezing procedure significantly scrambling the ensembles of conformations obtained in the freely folding stage (as shown by the right panel in Figure S6, Supporting Information), the RNA2 seemingly keeps track of a few robust secondary motifs throughout the encapsidation process, in a strongly salt‐dependent manner.

### 3.3 On the Search for an in Silico Match to the Experimental Observables

A further effort was ultimately devoted to exploring the diverse MD setup choices, in an attempt to target the global observables reported by Marichal et al.^[^ ^49^ ^]^ upon the equilibrium behavior of RNA2 in solution. As observed earlier, strengthening the electrostatic shielding via the effective salt concentration (0.15–0.5 M) arguably deals a major contribution to the (stabilization of the) gyration radius of the freely‐folding RNA2. Thus, a subsequent enhancement was taken by lowering the temperature to the state point of 293 K adopted in the experimental setup. While driving the system closer to the target value of *R*_*g*_ ∼ 10 nm (the experimental uncertainty, however, not being reported by the authors), thereby increasing the amount of hydrogen bonds with respect to the MD replicas at 310 K (see Figure 4), still the configurational ensemble sampled by the RNA2 molecule might be somewhat off‐target. In fact, we noticed that the distances between the 3' and 5' termini of the freely folding RNA2 do not comply with the expected values of a few nanometers reported for naturally‐occurring RNA molecules.^[^ ^44^ ^]^

Hence, to verify whether the end‐to‐end distance impacted on the behavior of RNA2, we setup a further MD scenario at 0.5 M and 293 K where the 3' and 5' termini were harmonically constrained within about 4 nanometers from one another (see Section 2.2 in the Experimental Section)—thereby enforcing an a priori bias to effectively depict a spontaneous behavior of RNA2.

In fact, the application of a mild harmonic restraint contributes a further decrease of the radius of gyration of RNA2 to *R*_*g*_ = 13.29 ± 0.68 nm (average over 3 replicas), ultimately approaching the experimental value and the inner radius of the capsid—as shown by Figure 4. Notably, this effect is not accompanied by an increase in the amount of hydrogen‐bonding contacts (see Figure 4) nor it accounts for a significant re‐framing of the secondary/tertiary motifs—if only for a single pseudoknot shown within a single MD replica (see Figure S9, Supporting Information).

As for the subsequent squeezing and encapsidation stages, which were carried out by keeping the harmonic restraint on the RNA2 termini, Figures S25 and S26 (Supporting Information) recapitulate the outcome of the scenarios in Section 3.2: In fact, while slightly lowering the amount of intramolecular pairings with respect to the freely folding MD, the embedding within a CCMV‐like capsid yields a rich variety of pseudoknots and long‐range motifs—arguably on account of the spatial confinement and enhanced density of nucleotides about the inner capsid walls. Furthermore, it is worth noticing that relieving the harmonic potential about the RNA2 termini throughout the encapsidation stage yielded a similar outcome in terms of the amount of hydrogen‐bonding interactions and stable secondary/tertiary motifs (i.e., pseudoknots and long‐range interactions).

## 4 Conclusion

In this work, we have explored the folding dynamics and conformational ensemble of the RNA2 fragment of the cowpea chlorotic mottle virus, by means of CG molecular dynamics and mean‐field approaches that explicitly take into account the electrostatic environment of the inner cavity of the capsid.

In the freely‐folding context, diverse MD replicas converge to consistent values of the gyration radii and amount of hydrogen‐bonding interactions. In fact, the RNA2 displays a dynamical equilibrium at both 0.15 M and 0.5 M, whereby the distribution of nucleotide pairings and secondary structures swiftly varies throughout the MD trajectories. Notably, the RNA2 molecule consistently relies on an array of short‐ranged, stable contacts – found in all MD replicas at 0.15 M and to a lesser extent at 0.5 M — regardless of the starting conformation. Yet, little corrspondence has been observed between MD and the contact map associated with the minimum free energy structure predicted by the RNAfold software of the ViennaRNA suite^[^ ^43^ ^]^ (between 5% and 15%, see Figure S10, Supporting Information). In principle, this discrepancy might be ascribed to the inherent limitations of either technique and of the force field, thereby requiring further scrutiny.

A significant mismatch was initially observed between the gyration radii of the RNA configurations obtained by freely folding MD and the available experimental estimates of Marichal and co‐workers:^[^ ^49^ ^]^ This was bridged effectively by i) enhancing the shielding of the Debye–Hückel electrostatics, ii) matching the experimental temperature of 293 K, and iii) enforcing a mild harmonic potential that restrained the RNA termini within a few nanometers from one another. Yet, the (somewhat consistent) absence of pseudoknots and long‐range tertiary motifs in the conformational ensembles of the freely folding RNA2 scenarios might highlight a few inherent limitations of the oxRNA force field here employed ‐ which in fact lacks an explicit treatment of the saline medium and a proper description of several non‐canonical interactions.

Subsequently, a two‐steps encapsidation protocol was followed: first, we confined the RNA2 molecules into a spherical volume compliant with the inner cavity of CCMV; second, we allowed the RNA to adjust to the electrostatic field within the capsid. For the latter, two mean‐field approaches were adopted, based on either the theoretical formalism reported by Šiber and Podgornik^[^ ^53^ ^]^ (analytical approach), or derived from a static depiction of the atomistic structure of the inner walls and N‐terminal tails of CCMV and the ionic distribution thereof (structure‐based approach).

Despite these alternative formulations achieving somewhat analogous potential profiles, their impact on the behavior of the encapsidated RNA2 differs substantially. In fact, the analytical approach wipes all traces of secondary structures and nucleotide pairings obtained in the freely folding scenario, whereas the structure‐based approach significantly shuffle the contact pattern of RNA2 while yielding a rich variety of medium‐ to long‐range motifs and pseudoknots. This is in line with ref. [74], where different secondary structures of packaged and free RNA have been experimentally observed in STMV. Yet, a significant fraction of the stable contacts observed at 0.15 M is found in all MD replicas of both the free and the encapsidated scenarios, thereby highlighting a critical role of the saline medium to driving sequence‐specific and kinetic effects. Although further scrutiny is in order, these results overall remark that the variability of secondary structures is substantial in both regimes.^[^ ^26^, ^27^, ^28^, ^31^ ^]^

Arguably, the description of the CCMV electrostatic field, which enforces a significant bias upon the conformational ensembles explored by RNA2, is critically sensitive to the charge distribution of the inner capsid walls and the ionic shells thereof.^[^ ^4^, ^21^, ^22^ ^]^ To this concern, our results corroborate the hypothesis that a proper description of the electrostatic contribution from the N‐terminal tails of the capsid, even only at a mean‐field level, is required to achieve stable RNA secondary structures *in virio*. This is in line with earlier CG simulations of Perlmutter et al., who inferred that viral RNA is overcharged by relying on the explicit inclusion of the charge carried by the capsid tails.^[^ ^21^ ^]^

We remark that, by employing effective approaches, we are limited in our capability to infer about the actual folding and packaging pathways of RNA2: In fact, both processes are influenced by a variety of co‐factors in vivo and in cultured cells experiments,^[^ ^14^ ^]^ although CCMV was demonstrated to spontaneously yield viral assemblies in vitro that are indistinguishable from the native, infectious particle.^[^ ^75^ ^]^ Moreover, the two‐step, in vitro assembly of the CCMV virions likely follows an en masse‐adsorption mechanism,^[^ ^10^, ^76^ ^]^ whereby a stoichiometric excess of capsid subunits is reversibly adsorbed onto the RNA chain, only to coalesce into a fully‐mature virus‐like particle upon lowering the pH. This latter aspect is critical as the CCMV displays diverse morphologies upon varying the pH and the (mono, divalent) salt concentration—both of which modulate the interactions between capsomers and with the RNA. In fact, the structural variety of the CCMV assemblies—and transitions thereof—has been the subject of extensive research, both in vitro and in silico,^[^ ^55^, ^75^, ^77^, ^78^, ^79^ ^]^ and, despite lying out of the scope of this work, it highlights a limitation of our approach and of the oxRNA2 force field in their current implementation.

This notwithstanding, numerical techniques might be of aid in the characterization of viral RNAs: For instance, with no a priori knowledge on the encapsidated RNA structures, Larsson and van der Spoel have inferred that the in silico distribution of chloride ions well‐recapitulates the electron density of the ssRNA in STMV and STNV,^[^ ^80^ ^]^ thereby implicitly suggesting that a proper effective potential should be derived via a self‐consistent protocol, taking into account the very distribution of the RNA molecule. Similarly, this work demonstrates the capabilities of the numerical tools at our disposal to characterize the inherent structural variability of viral RNAs. Moreover, the quantitative description of a capsid‐like environment via the combination of molecular dynamics with a multi‐scale modeling approach defines an effective framework taking into account the explicit contribution of the electrostatic forces at an apt level of resolution: In fact, this protocol is broadly applicable to single‐stranded RNA viral genomes of any length, for which the sequence and 3D structure of the proteic capsid shell is known. As such, it might support the conventional approaches based on thermodynamic structure predictions, in the perspective of a proper mechanistic depiction of the assembly process.

## Conflict of Interest

The authors declare no conflict of interest.

## Author Contributions

G.M. and M.M. performed all the (atomistic, CG) MD simulations and the data analysis thereof, and wrote parts of the MS. L.P. aided in performing the atomistic MD simulations, constantly supervised and coordinated the project, and contributed majorly to the writing of the MS. L.R. performed the annealing simulations and the data analysis thereof, oversaw and coordinated the project, and wrote parts of the MS. L.T. oversaw and coordinated the project, and contributed significantly to the writing of the MS. S.P. and R.P. oversaw, funded and coordinated the project and contributed to the writing of the MS.

## Supporting information

Supporting Information

## Data Availability

The data that support the findings of this study are openly available in Zenodo at https://zenodo.org/records/14058158, reference number 14058158.
