# Supplementary material for: Molecular Dynamics Characterization of the Free and Encapsidated RNA2 of CCMV with the oxRNA Model
Source: Macromol Rapid Commun. 2024 Nov 22;45(24):2400639. doi: 10.1002/marc.202400639 (PMC11661665; doi:10.1002/marc.202400639)
Supplement: Supplementary file 1 — Supporting Information [file MARC-45-2400639-s001.pdf]

Macromolecular Rapid Communications

Supporting Information

for *Macromol. Rapid Commun.*, DOI 10.1002/marc.202400639

Molecular Dynamics Characterization of the Free and Encapsidated RNA2 of CCMV with the oxRNA Model

*Giovanni Mattiotti, Manuel Micheloni, Lorenzo Petrolli, Lorenzo Rovigatti, Luca Tubiana, Samuela Pasquali and Raffaello Potestio**

Supporting Information
Molecular dynamics characterization of the free and encapsidated RNA2 of CCMV with the oxRNA model

Dr. Giovanni Mattiotti,[1] Manuel Micheloni,[2,3] Dr. Lorenzo Petrolli,[2,3] Prof. Lorenzo Rovigatti,[4] Prof. Luca Tubiana,[2,3] Prof. Samuela Pasquali,[1] and Prof. Raffaello Potestio[2,3]

[1]*Laboratoire Biologie Functionnelle et Adaptative,*
CNRS UMR 8251, Inserm ERL U1133,
Université Paris Cité, 35 rue Hélène Brion, Paris, France
[2]*Department of Physics, University of Trento,*
via Sommarive, 14 I-38123 Trento, Italy
[3]*INFN-TIFPA, Trento Institute for Fundamental Physics and Applications, Trento, Italy*
[4]*Department of Physics, Sapienza University of Rome,*
p.le A. Moro 5, 00185, Rome, Italy

I. RNA2 SEQUENCE

1 GUAAUCCACG AGAGCGAGGU UCAAUCCCUU GUCGACUCAC GGGUCUCCAU CAGUUGAAAA
61 CAGUUUAUAC AUUUUCUUCU UGAUAUUUUU CUUCUUUACU UCCAUUAAUA UGUCUAAGUU
121 CAUUCCAGAA GGUGAGACUU ACCACGUUCC CUCAUUCCAA UGGAUGUUUG AUCAGACUCU
181 CGAAUCUGAC UCACACCAUG AUGAGGCGAU AUUCGUAACC GAAUCGAUUA AUGAAAGUGG
241 AGUUGAUACU UCUGUUGAAA UAACCGCAGA UGGCACGCUA GCAAGUUAUA UGCAUGCCGU
301 AAAGCCCCUA GUGGAGGAUG GUCUUCUGAA UCCCCCUUUU GAUCAAGCUA GAUGGGGUCU
361 UUGCUGCAAG AACGUCGUUG ACGUUUAUGA CGGGCUGCUC GGUUAUAGAC UCAUACCAAU
421 GGCUGAAGCC GCUAGAAUGU UGUACUUGGA AAUCGACGGU UCAUUCGUUG AUGAAUCUGA
481 GUGUGACGAU UGGCGGCCGG UAGAUACCUC UGAUGGUUUC ACCGAAGCAA UGUUUGAUGU
541 GAUGAAUGAG AUUCCUGGCG AGGAAACAAA AAAUACAUGC GCUUUAAGUC UUGAAGCUGA
601 AUCAAGGCAA GCUCCAGAAA CUUCCGAUAU GGUGCCGUCU GAAUAUACGU UGGCAGAUAG
661 GUACGUUACC ACCAGAGAGG AGUUCGCGUC UGUUGACUCG GAUUAUGACA UAUCCUUAAA
721 CCUGGUGAGC CCUGUGGAGU UCAGGGUGGG AGUGUGUGAA GACACAUACC GUCAUUCGGA
781 AGCUGAUGAU CCUACGAUGC CUCAAUAUCA CGAUAGGAUC AGUUUAAAAU CGCUGGAGGC
841 GGCUGGCCAU CACAUGUUAC CGACUCAUGC CUAUUUUGAC GACACUUACU ACCAGGCUUU
901 GGAAGAGCUA GGCGAUUAUA AUGUCGAUAU UAGUAAGUUG UCUGUCCGGC AGAGUGAUGU
961 UGAUUGGUAU CGUGACCCUG AAAAGUACUA UGAGCCUGAG UUAAGUAUAG GGUCAUUCCA
1021 ACGUAGAAUA GGUACGCAAA AGACGGUCCU UACCGCGUUA AAGAAACGGA ACGCUGACGU
1081 GCCUGAGUUA GCAGAUUCUG UUGAUAUUAA AAGAGUAGCC UGUGAAGUAG CUGAAAAAUU
1141 UAAACGGGCU UAUCUUAAUC AUUCCGGUAU AGGGCUGUUA GGGCAAAGUA UGGAUGUCAU
1201 GUCCAGAGGA CUUGAGUACC AUAAGAAAUG GAAAGACCAC AAAGACCUGA CUGGUGUGAC
1261 AGUUUUGUCU GAGAUUAAUU UGCAGAGGUA UCAGCACAUG AUAAAGUCUG AUAUUAAACC
1321 AGUUGUCUCG GAUACGUUAC ACCUCGAACG AGCUGUUGCU GCAACAAUAA CAUUUCAUGG
1381 UAAAGGAGUU ACUAGCUGCU UCUCACCAUA UUUUACGGCU UGUUUCGAGA AGUUUUCAAA
1441 AGCUUUAAAA UCAAGGUUUG UGGUCCCCAU AGGGAAGAUC UCCUCCCUGG AACUGAAAAA
1501 UGUUCCCCUC UCGAAUAAAU GGUUUCUUGA GGCGGAUUUG AGUAAGUUUG AUAAAUCUCA
1561 GGGUGAGCUU CAUCUUGAGU UCCAAAGAGA GAUAUUGUUG UCAUUGGGUU UUCCAGCCCC
1621 UUUGACUAAU UGGUGGUGUG AUUUCCAUAG GGAAUCUAUG CUAUCGGAUC CUCAUGCUGG
1681 AGUUAACAUG CCAGUUUCCU UUCAGCGUCG UACUGGUGAU GCUUUUACUU AUUUUGGGAA
1741 UACUUUGGUG ACUAUGGCCA UGAUGGCCUA UUGUUGCGAU AUGAACACCG UGGACUGUGC
1801 UAUCUUUUCC GGUGAUGAUU CUCUGUUAAU UUGUAAAAGU AAACCACAUC UGGAUGCUAA
1861 UGUUUUUCAA UCUCUGUUUA AUAUGGAAAU UAAAGUUAUG GACCCAAGUU UGCCAUACGU
1921 UUGUAGUAAG UUUCUUUUAG AAACUGAAAU GAAUAACUUG GUGUCUGUGC CUGAUCCUAU
1981 GAGAGAGAUA CAGAGACUGG CUAAGCGAAA GAUCAUCAAA UCGCCUGAGU UGUUAAGAGC
2041 CCACUUUGAG UCCUUUUGUG AUAGGAUGAA AUUCCUAAAC AAAUUGGAUG AAAAAAUGAU
2101 AAAUUUAUUA UGCAAGUUUG UGGCUCUCAA GUAUAAAAAA CCUGACGUUG AAAACGAUGU
2161 CAGAGUAGCC AUUGCUGCUU UCGGCUACUA CUCAGAAAAU UUCUUGAGAU UUUGCGAAUG
2221 UUAUGCGACU GAAGGGGUCA AUAUAUAUAA GGUAAAACAU CCCAUCACCC AGGAGUGGUU
2281 CGAGGCCUCU AGGGAUCGAG ACGGUGACUG GUUCCAUGAC UGGCGUAAUC CGAAGUUUCC
2341 CACUGCCUUA GAUAAGGUUU GGAGAUUCUU UGGAAAAUAC GCGAGAGAUG AUCCUAUGAA
2401 GCACAUAGAA GAGAGAGAUA GGAGACAUAG GCUUAAUCGA GCCAUGAAUU CUUCCUUGAA

2461 ACUUGCCUAU GAUCGUAGGA GUCUUAGUAA GGAUAAAGAA ACCGUUGCGU GGGUGCGUAA
2521 GACCCUUUCU AAAUAAUGUU GGUCACAUUU AAGACUUGUU UAGUCCACAU UAGGACUGGU
2581 UCUAACAGUU UCUUUAAACU GUAAUCGUCG UUGCGACGUU GGUUUGCUUA CAAGCAAUCA
2641 AGCUGCCUUU GAGUUUUACU CCUUGAACUC UUCAGAAGAA UUCUUCGGAA UUCGUACCAG
2701 UAUCUCACAU AGUGAGGUAA UAAGACUGGU GGGCAGCGCC UAGUCGAAAG ACUAGGUGAU
2761 CUCUAAGGAG ACCA

II. FREELY-FOLDING STAGE

A. RNA2 conformations

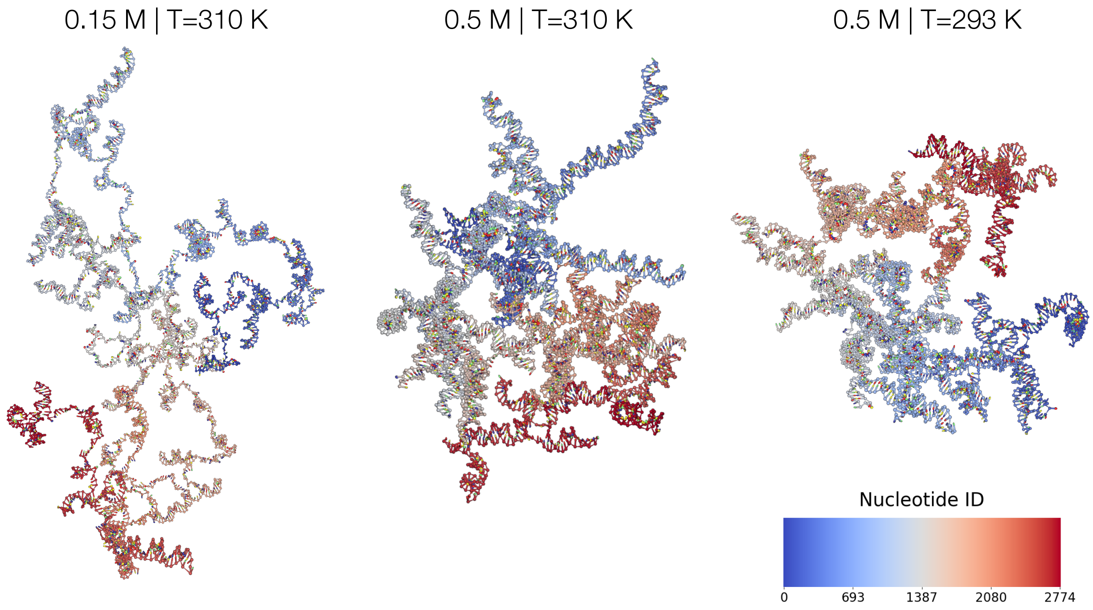

Supplementary Figure S1: (Example) snapshots of RNA2 conformations at (**left**) 0.15 M ($T = 310$ K), (**center**) 0.5 M ($T = 310$ K), and (**right**) 0.5 M ($T = 293$ K, the RNA termini fixed by harmonical restraints), as a result of the freely-folding MD. The color code is associated with the nucleotide index.

B. Distance matrices between (intra-replica, intra-concentration) contact maps

To quantify the variability in the secondary structures of RNA2, we defined a distance d_{KM} between contact maps (called KM) as follows: Given two Boolean contact maps $\{k_{ij}^{(1)}\}$, $\{k_{ij}^{(2)}\}$ associated with frames (1) and (2), the distance counts all contacts that are "true" in either (not both) frames - which is equivalent to a logical XOR criterion. The 2D matrices showing the values of d_{KM} for each duple of frames within a given replica, and within all MD replicas per salt concentration, are shown in Figure **S2**. A hierarchical clustering based on an *average linkage* criterion was performed on the (intra-concentration) distance matrices of Figure **S3** - dendrograms are shown in Figure **S4** (0.15 M) and **S5** (0.5 M).

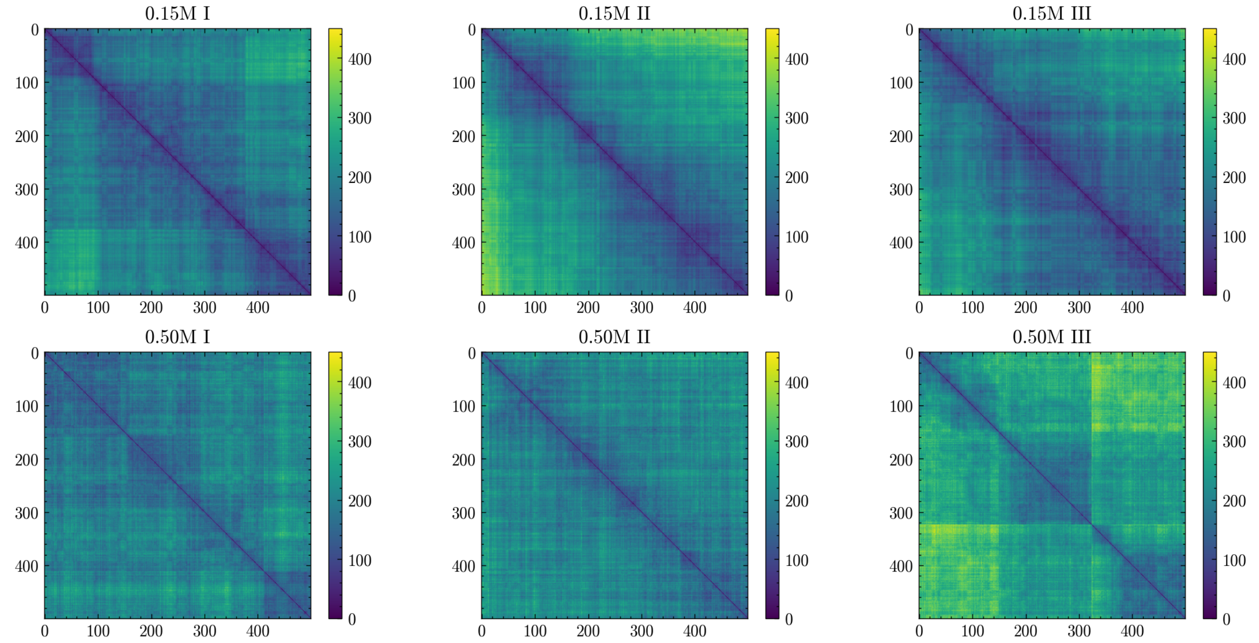

Supplementary Figure S2: 2D distance matrices (d_{KM}) between contact maps, associated with the freely-folding MD replicas at 0.15 M (top row) and 0.5 M (bottom row).

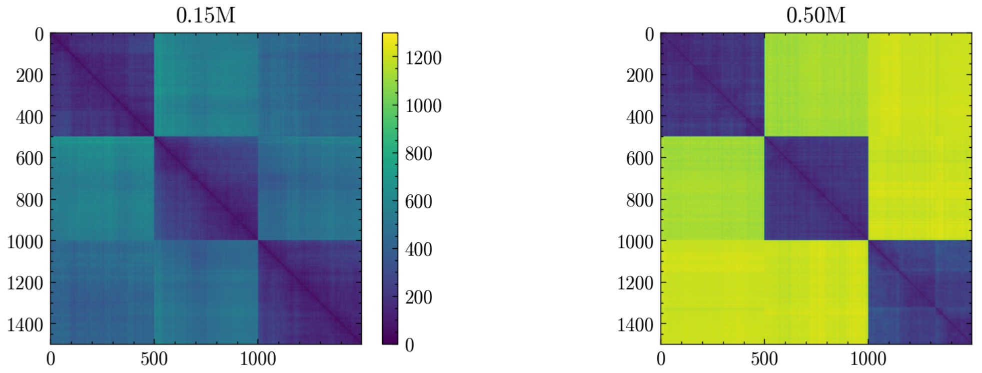

Supplementary Figure S3: 2D distance matrices (d_{KM}) between contact maps, associated with all frames from all replicas of the freely-folding MD at (**left**) 0.15 M and (**right**) 0.5 M.

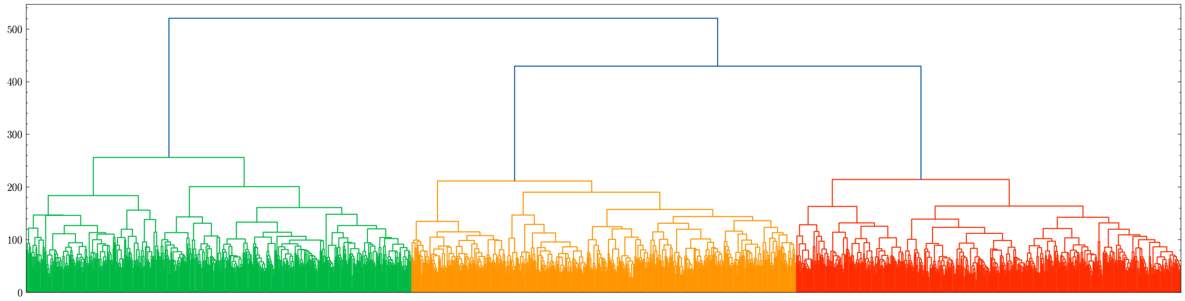

Supplementary Figure S4: Dendrogram depiction of the hierarchical clustering of frames from all MD replicas at 0.15 M, based upon the distance between contact maps together with an average linkage criterion: A 1:1 association between MD replicas and clusters highlights the structural dissimilarity between conformational ensembles.

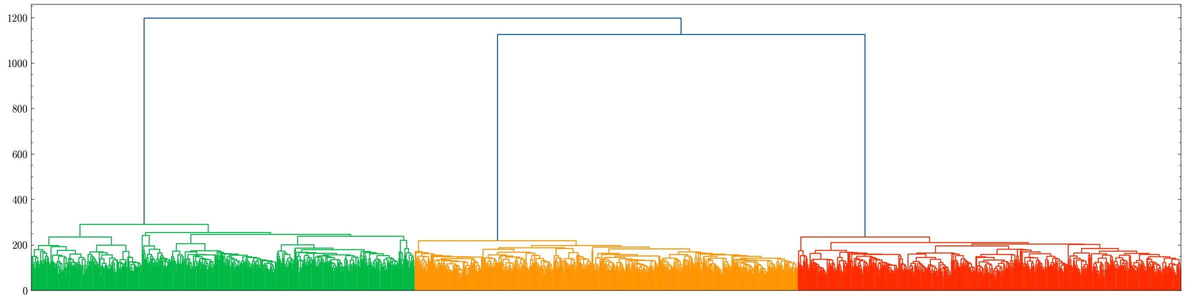

Supplementary Figure S5: Dendrogram depiction of the hierarchical clustering of frames from all MD replicas at 0.5 M, based upon the distance between contact maps together with an average linkage criterion: A 1:1 association between MD replicas and clusters highlights the structural dissimilarity between conformational ensembles.

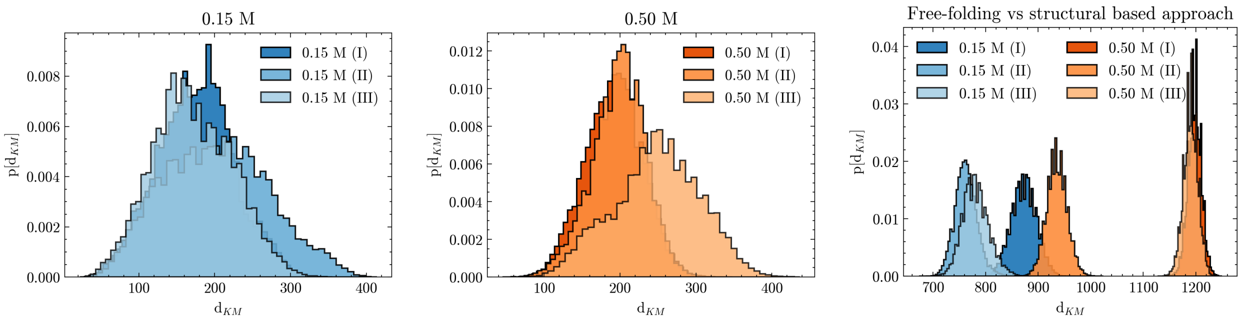

Supplementary Figure S6: Normalized histograms of the distances between contact maps (as described in the "Analysis protocol" section), the latter being collected on a frame-wise basis for each replica of the freely-folding MD at 310 K and (**left**) 0.15 M or (**center**) 0.5 M. **(Right)** Normalized histograms of the all-v-all, cross-distances between the contact maps from the freely-folding MD at 310 K and the contact maps from the dynamics of RNA2 within the CCMV-derived external potential: The "shuffling" of the secondary/tertiary structures associated with the encapsidation procedure is highlighted.

C. Free energy landscapes

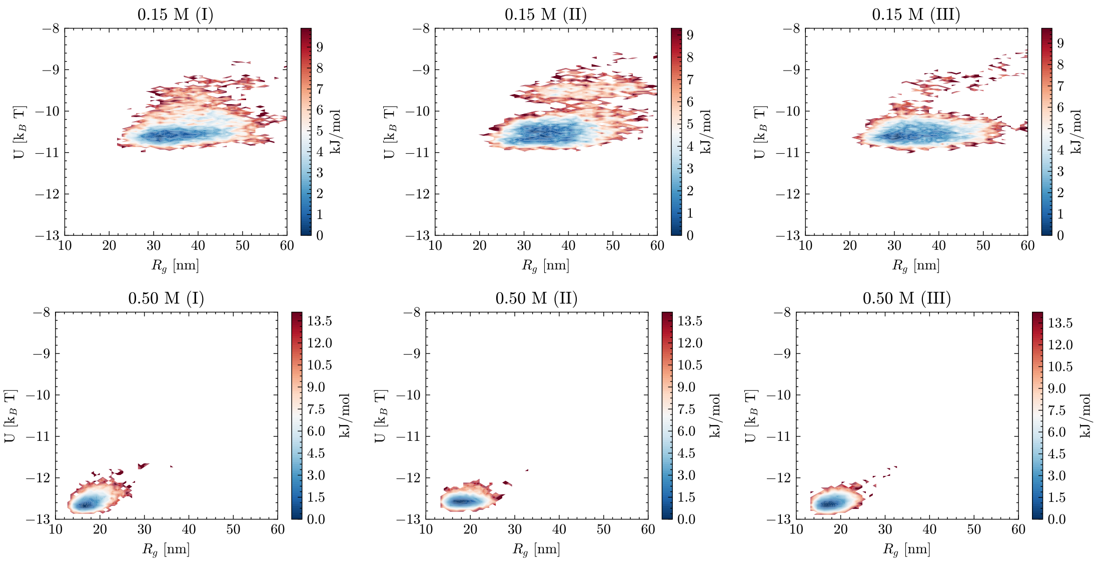

Supplementary Figure S7: Free-energy profiles of the RNA2 from the freely-folding MD replicas at (**top**) 0.15 M, and (**bottom**) 0.5 M, based upon the values of the gyration radius (R_g) and the internal energy (U).

D. Chord diagram depiction of the stable hydrogen-bonding contacts

1. *MD simulations of the RNA2 with with unconstrained termini at T=310 K*

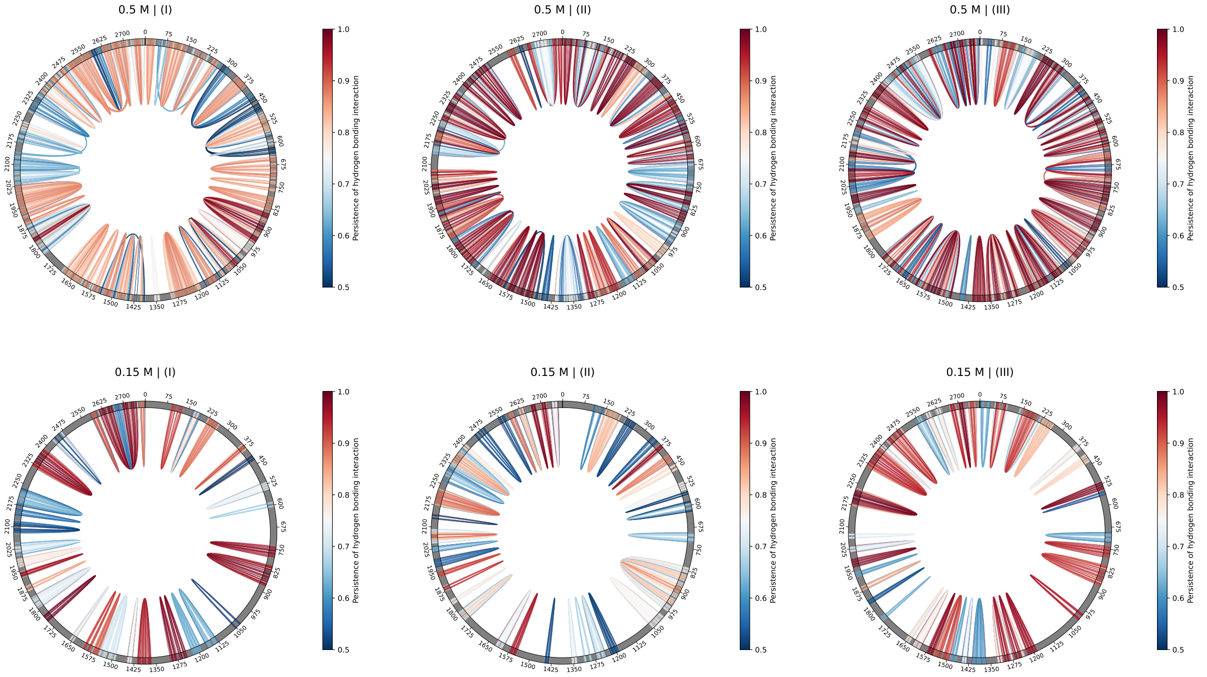

Supplementary Figure S8: Chord diagram illustrating the stable hydrogen-bonding contacts, defined as those conserved in over 50% of trajectory frames across independent MD replicas. The top row shows results from simulations at 0.5 M, while the bottom row displays 0.15 M.

2. *MD simulations of the RNA2 with harmonically constrained termini at T=293 K*

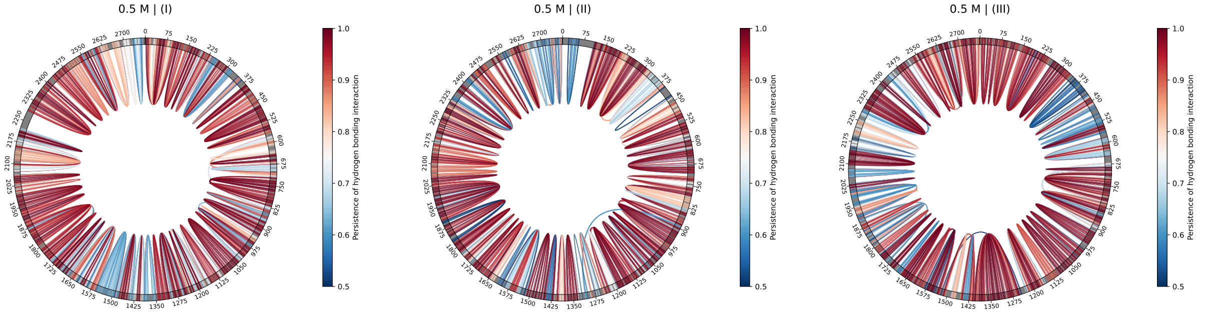

Supplementary Figure S9: Chord diagram depiction of the stable hydrogen-bonding contacts, i.e., conserved in over 50% of the trajectory frames, over the equilibrated fraction of (**upper left**) replica I, (**upper right**) replica II, and (**bottom**) replica III of the freely-folding MD at 0.5 M at $T = 293$ K.

E. Similarity between the contact maps achieved by freely-folding MD and the (optimal) RNAfold structure prediction

Firstly, we obtained the optimal secondary structures prediction by the RNAfold software of the ViennaRNA suite [1], according to the appropriate conditions:

- $T = 310$ K, $I = 0.15$ M: total amount of HB interactions = 874
- $T = 310$ K, $I = 0.50$ M: total amount of HB interactions = 878
- $T = 293$ K, $I = 0.50$ M: total amount of HB interactions = 899

We thus calculated the amount of HB contacts that are shared between each frame of (the equilibrated fraction of) the 12 replicas from the 4 freely-folding MD scenarios and the corresponding RNAfold prediction (as shown by the histogram in **Figure S10**).

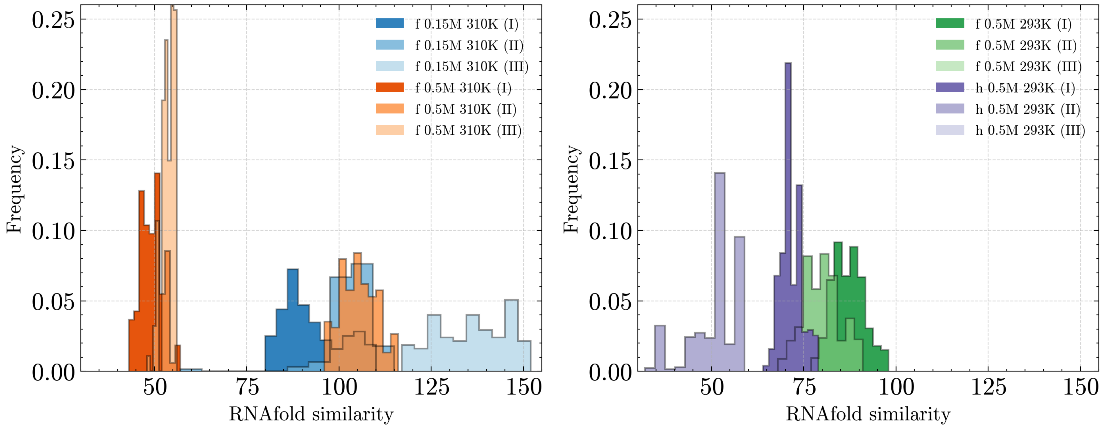

Supplementary Figure S10: Normalized histograms of the number of contacts shared between each frame of the freely-folding MD replicas at (**left**) $T = 310$ K, and (**right**) $T = 293$ K) and the optimal 2D structure prediction obtained by RNAfold - according to the appropriate experimental conditions.

As a reference, we hereby report an approximate estimate of the average number of hydrogen-bonding interactions per each of the 4 simulated scenarios:

- $T = 310$ K, $I = 0.15$ M: aver. amount of HBs $\simeq 470$
- $T = 310$ K, $I = 0.50$ M: aver. amount of HBs $\simeq 640$
- $T = 293$ K, $I = 0.50$ M: aver. amount of HBs $\simeq 760$
- $T = 293$ K, $I = 0.50$ M, constrained RNA termini: aver. amount of HBs $\simeq 750$

F. End-to-end distance of the RNA2 fragment during the freely folding stage

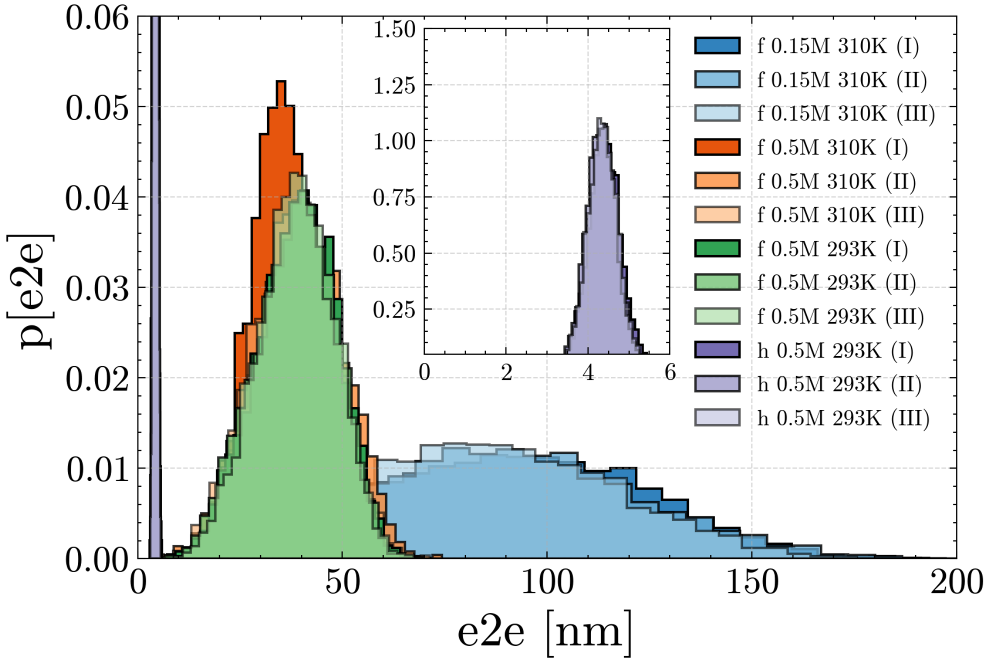

Supplementary Figure S11: Normalized histograms of the end-to-end distances between the 3' and 5' termini of the RNA2 fragment, calculated frame-wise along each replica of the four freely-folding MD scenarios.

G. Freely-folding simulations *via* the annealing protocol

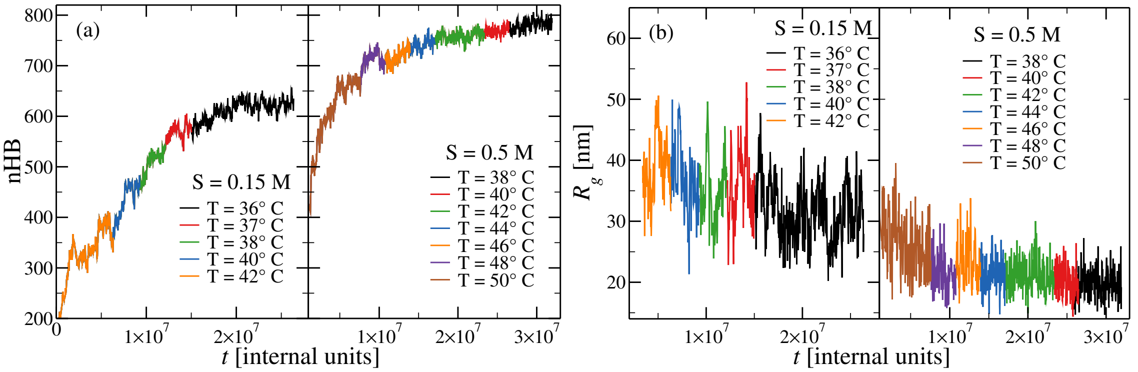

Supplementary Figure S12: (**a**) The number of hydrogen bonding interactions, and (**b**) gyration radii of the RNA2 fragment as function of the simulation time, obtained *via* the annealing protocol. The left and right sub-panels of each figure refer to a salt concentration of 0.15 M and 0.5 M respectively.

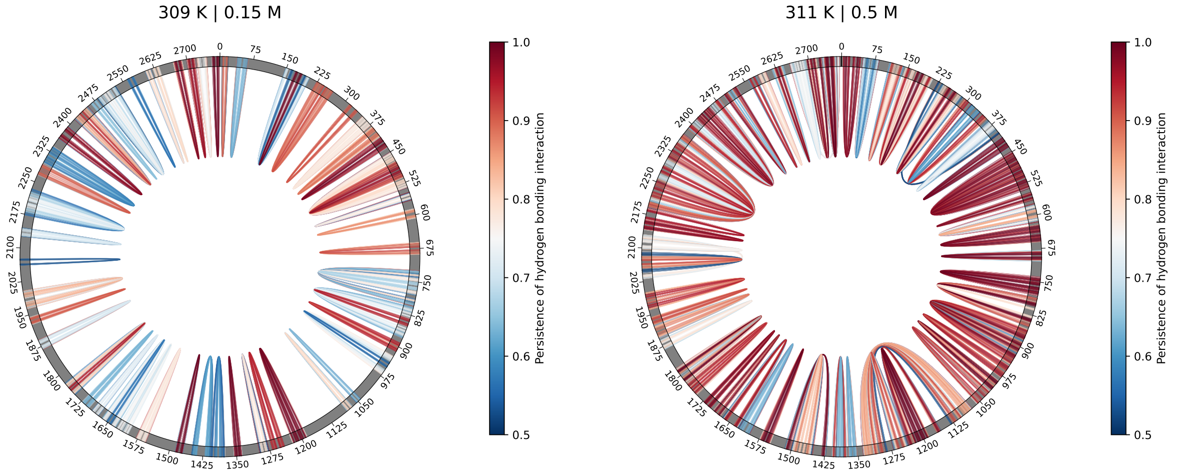

Supplementary Figure S13: Chord diagram depiction of the stable hydrogen-bonding contacts, i.e., conserved in over 50% of the trajectory frames, collected in the last fraction of the annealing protocol for the two saline concentrations (black lines in Figure **S12**).

In addition to the free-folding simulations, we have attempted to fold the RNA2 *via* an annealing protocol, thereby monitoring the number of hydrogen-bonding interactions (nHB) and the gyration radius (R_g) as the temperature is decreased over time. For both salt concentrations adopted (0.15 and 0.5 M), simulations were started at a temperature where the RNA showed no secondary structure, thus decreasing T in discrete steps of 1 or 2 K, until both nHB and R_g converged to a plateau. Each stage of the simulation (i.e., at a constant temperature) took 1-4 days of GPU time.

Figure **S12** shows the evolution of both quantities as a function of the simulation time: In fact, the annealing protocol does not seemingly yield better (*i.e.*, smaller) values of the R_g than those obtained *via* free-folding MD, despite achieving RNA2 structures with a slightly

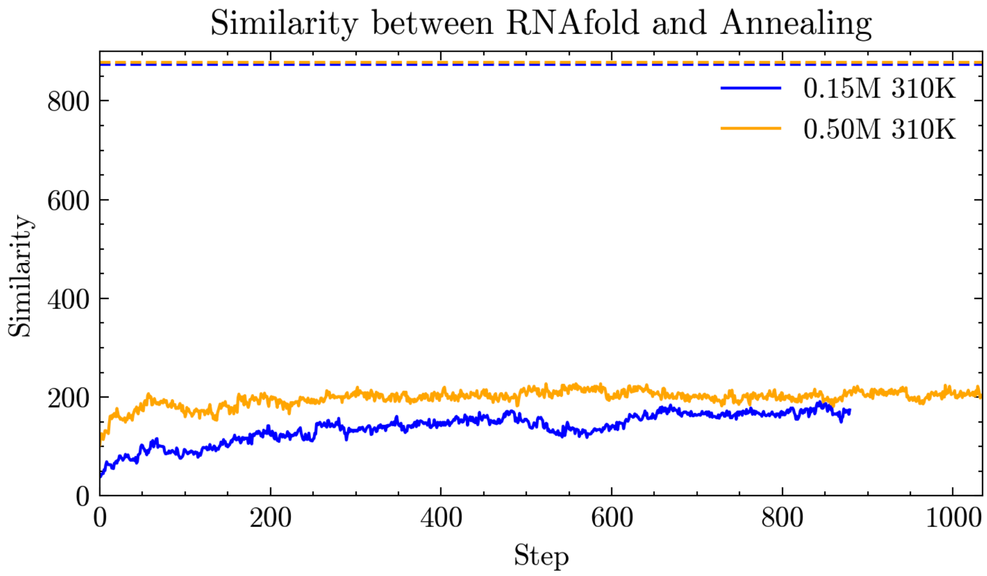

Supplementary Figure S14: Similarity between the RNA2 secondary structures obtained by the annealing protocol and the MFE prediction of RNAfold - expressed as number of base pairings shared by both techniques - at 310 K and a 0.15 M/0.5 M monovalent salt concentration. The total numbers of base pairs predicted by the RNAfold MFE are shown as dashed lines.

($\approx$ 20%) higher number of hydrogen bonds.

The similarity between the secondary structures yielded by the annealing protocol and the one associated with the MFE structure of RNAfold is somewhat higher than that obtained by the freely-folding MD protocol (as shown by **Figure S14**). Yet, these values do not exceed 12.5% (0.15M) and 25% (0.50M) of the total base pairings, remarking a significant difference between the oxRNA and the RNAfold predictions.

III. ENCAPSIDATION STAGE

A. Evolution of the internal pressure and of the hydrogen-bonding interactions

We monitored the internal pressure of the RNA2 molecules throughout the encapsidation stage (see Equation **1** in the main text), as effective proxy of a quasi-static transformation. According to this criterion, the RNA replicas at 0.5 M, T = 310 K, and unconstrained termini, were somewhat unaffected by the external force, associated with an encapsidation kinetics of 5.6×10^{-8} λ_{ox}/MD steps (see Figure **S15**) - eventually yielding a spherical volume about 10 nm in radius. As for the simulations at 0.5 M, T = 293 K, and harmonically-constrained termini, the encapsidation rate was kept, while the final radius was set about 11 nm.
Conversely, the encapsidation of the RNA2 at 0.15 M required that a milder kinetics of 1×10^{-8} λ_{ox}/MD steps be applied in the later stage, to keep the internal pressure from diverging - thereby achieving a spherical volume of about 11 nm in radius. Yet, as observed in

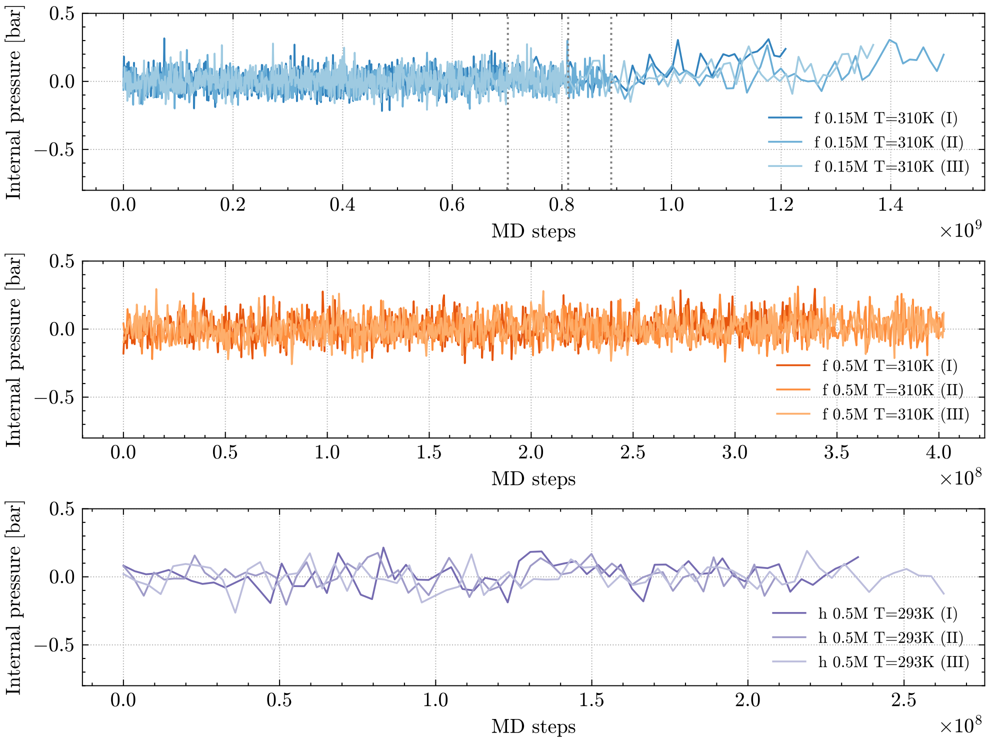

Supplementary Figure S15: Estimates of the internal pressure throughout the encapsidation stage of the RNA2. In particular, the inset at the top and center show simulation results performed at 0.15 M and 0.5 M, respectively, under conditions of $T = 310$ K and with unconstrained RNA ends (labeled as **f**). The bottom inset presents simulations performed at 0.5 M and $T = 293$ K, where the RNA termini are harmonically constrained (labeled as **h**).

Figure **S16**, the encapsidation procedure drives a significant rise in the amount of hydrogen-bonding nucleotides - which is steady at 0.5 M and abrupt at 0.15 M, yielding about a 60% contact increase in the latter case. While one might account for this process in terms of spatial proximity, forcibly favouring the pairing of nucleotides, we are inclined to believe this might be an artificial outcome from a strongly non-equilibrium setup.

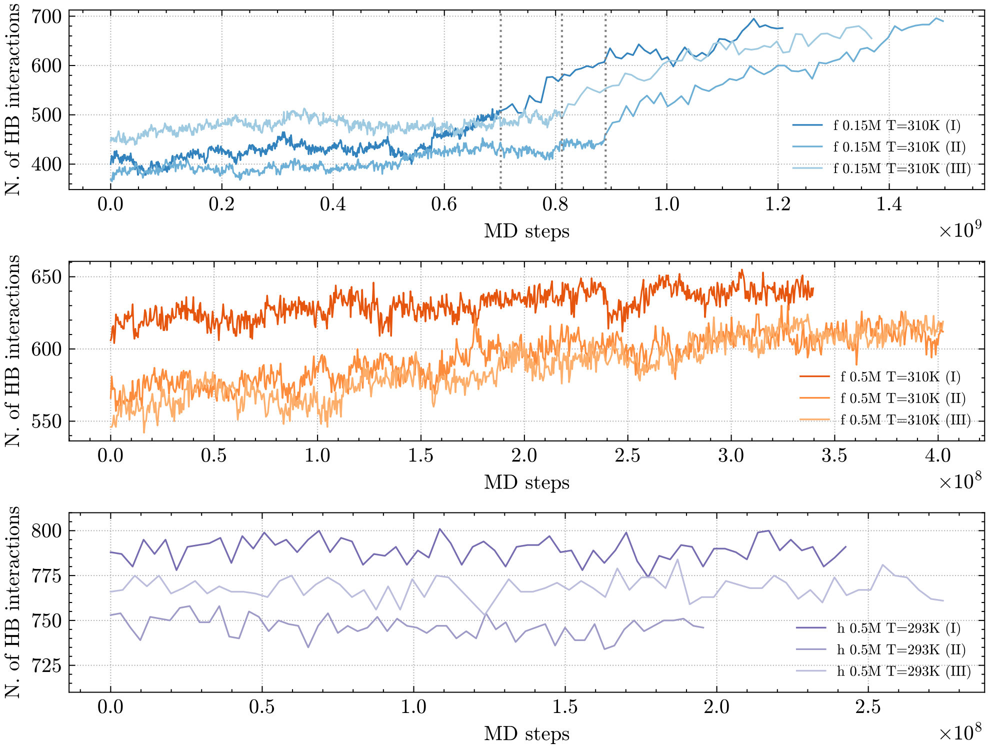

Supplementary Figure S16: Evolution of the amount of hydrogen-bonding interactions throughout the encapsidation stage of the RNA2. In particular, the inset at the top and center show simulation results performed at 0.15 M and 0.5 M, respectively, under conditions of $T = 310$ K and with unconstrained RNA ends (labeled as **f**). The bottom inset presents simulations performed at 0.5 M and $T = 293$ K, where the RNA termini are harmonically constrained (labeled as **h**)

IV. RNA2 DYNAMICS WITHIN A CCMV CAPSID-LIKE ELECTROSTATIC POTENTIALS

Two mean-field approaches were adopted to the calculation of a radial profile of the electrostatic potential, based upon the theoretical formalism reported by Šiber and Podgornik [2] and atomistic structural data respectively.

A. Derivation of a spherically-symmetric potential *via* an analytical approach

In Ref. [2], the capsid is depicted as a thin spherical shell of radius R_0, associated with a surface charge density $\sigma_c = Q/4\pi R_0^2$. Under the Debye-Hückel approximation of the Poisson-Boltzmann equation, an analytical solution for the electrostatic potential energy (of the internal cavity of the capsid) is thus derived as:

$$U_{theo}(r) = \frac{q_{nuc}\,\beta Q \sinh\left[k_{DH}(R_0 - r)\right]}{4\pi(R_0 - r)\epsilon_0\epsilon_r k_{DH} R_0 \{\sinh[k_{DH}(R_0 - r)] + \cosh[k_{DH}(R_0 - r)]\}} \tag{1}$$

with k_{DH} the inverse Debye length, Q the internal charge of the (amino-terminal tails of the) capsomer subunit at physiological pH ($+1800e$), $I = 0.15$ M the monovalent salt concentration, R_0 the internal radius of the CCMV capsid (about 12 nm). Here, we set the value of the probe charge q_{nuc} according to the effective charge of the single nucleotide in the oxRNA model.

Figure **S17** shows the outcome of a fitting procedure, whereby we redefined $U_{theo}(r)$ as a combination of a Yukawa (attractive) and WCA (repulsive) potential - refer to the main text for details.

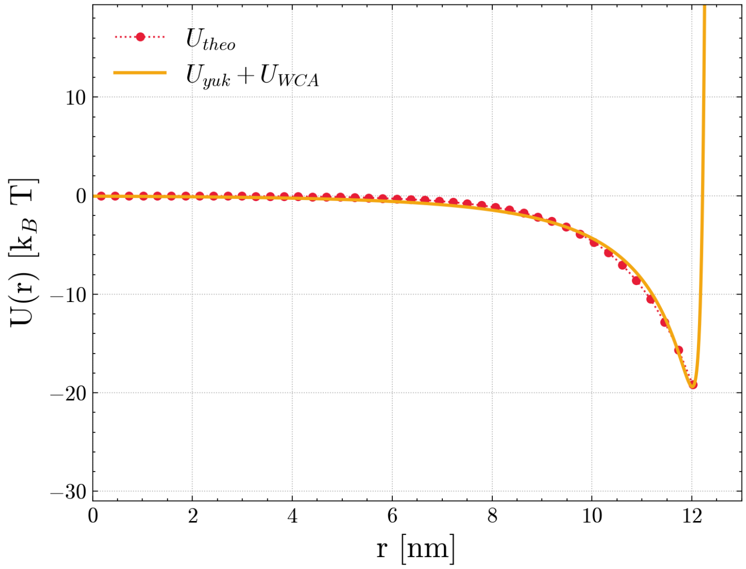

Supplementary Figure S17: Plots of the $U_{theo}(r)$ potential energy (red dots) and outcome of the fitting procedure $U_{yuk+WCA}(r)$ (orange solid line) - the latter employed as radial potential profile in the dynamics of RNA2 within a CCMV-like electrostatic field (analytical approach).

B. Derivation of a capsid-like external field from atomistic data

1. *Construction of a model capsid structure*

Coordinates of the trimer subunit of the CCMV capsid (chains A, B and C) were obtained from the RCSB Protein Data Bank (entry ID: 1CWP) [3]. The missing residues belonging to the amino-terminal tails of the capsomer subunit (that is, 26 amino acids of chains B and C, and 42 amino acids of chain A) were reconstructed *via* the Chimera visual interface [4] of the Modeller toolkit [5] - whereby the highest-ranking model of five alternative structures was kept.
The newly-achieved subunits were thus subject to a multi-step minimization/equilibration protocol[6], as follows:

1. a first energy minimization of the structure was carried out in vacuum, constraining all atoms of the monomeric subunits **but** the N-terminal tails;
2. the minimized structure was solvated and neutralized by an excess concentration (0.15 M) of sodium chloride;
3. by fixing all atoms of the capsomer subunits **but** the amino-termini, the capsid tails were further minimized and subject to subsequent equilibration steps in the NVT (1 ns) and NPT ensemble (200 ps);
4. from the latter step, we extracted the frame associated with the lowest radius of gyration and carried out a further (restrained) NVT run of $\sim$ 10 ns.

A minimized configuration of the trimeric capsomer is achieved, whereby we selected the coordinates of the monomeric subunit whose amino-terminus interfered the least with the neighboring capsomers. This structure was thus employed to reconstruct the whole CCMV capsid shell (*via* the `MatchMaker` function of Chimera):

1. firstly, by superimposing the subunit to each of the three chains of the trimeric capsomer;
2. hence, by replicating the trimeric capsomer upon the PDB template of the CCMV capsid shell, according to the sixty-fold symmetry of the icosahedral structure

2. *Energy minimization of the capsid structure and solvent thermalization*

The system topology of the newly-achieved capsid structure was initialized employing the CHARMM36m atomistic force field of biomolecular structures [7] - hence:

1. the capsid was firstly subject to a threefold minimization step in vacuum by a steepest descent algorithm, gradually lowering the force tolerance threshold (5000, 1000 and 100 kJ/(mol nm));

2. the vacuum-minimized system was solvated within a dodecahedral box of TIP3P water molecules and neutralized by an excess (0.15 M) concentration of sodium chloride - likewise, a three-fold minimization protocol was applied;

3. a 10-ps thermal annealing of the solvent bath (i.e., by fixing the coordinates of the CCMV capsid atoms, steadily increasing the temperature to 300 K) was thus performed, followed by a 1-ns thermalization of the solvent bath in the NVT ensemble.

Notably, this protocol yields a significant discontinuity in the radial distribution of the solvent medium, at the core of the capsid shell (as shown by Figure **S18**), despite the simulation showing no numerical instability. In fact, a similar artifact was observed earlier by Freddolino and co-workers [8], likely accounted for by a (slowly-equilibrating) mismatch between the density of the physiological medium (from the solvation routine) and that of the target system.

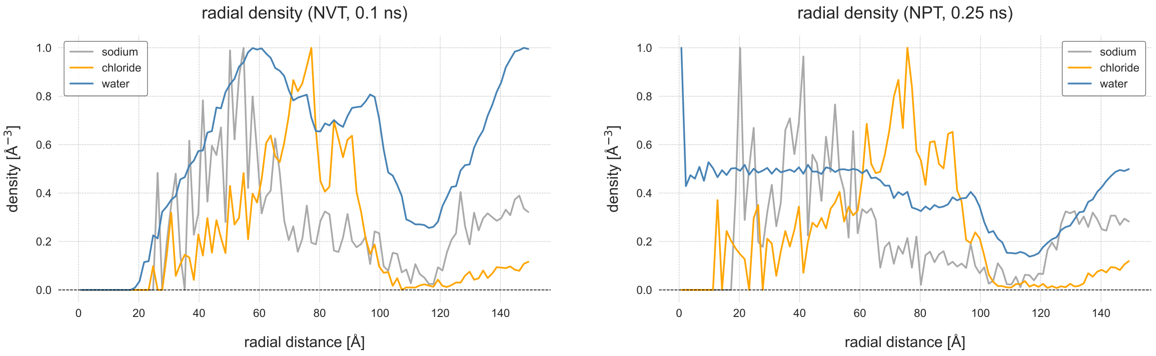

Supplementary Figure S18: Radial density profile of the solvent medium (**left**) after the first 100-ps thermalization in the NVT ensemble - highlighting the void at the core of the simulation box; (**right**) after the re-equilibration protocol (detail in the text). For the benefit of clarity, all values are normalized to 1.

The void was thus aptly filled *via* a further solvation step, and the capsid structure subsequently re-equilibrated by i) < 9000 steepest-descent, minimization steps (force threshold: 1000 kJ/mol nm); ii) a 500-ps solvent thermalization in the NVT ensemble; iii) a 250-ps density equilibration in the NPT ensemble.

3. Derivation of a radial potential profile from the atomistic structure of CCMV via Gauss' theorem

The distribution of the (partial) charges from the last frame of the MD minimization/thermalization protocol (described in Section IV B 2 - see Figure **S19**) was adopted in the calculation of a mean-field, radial electrostatic potential associated with the CCMV capsid and the electrolyte distribution thereof.

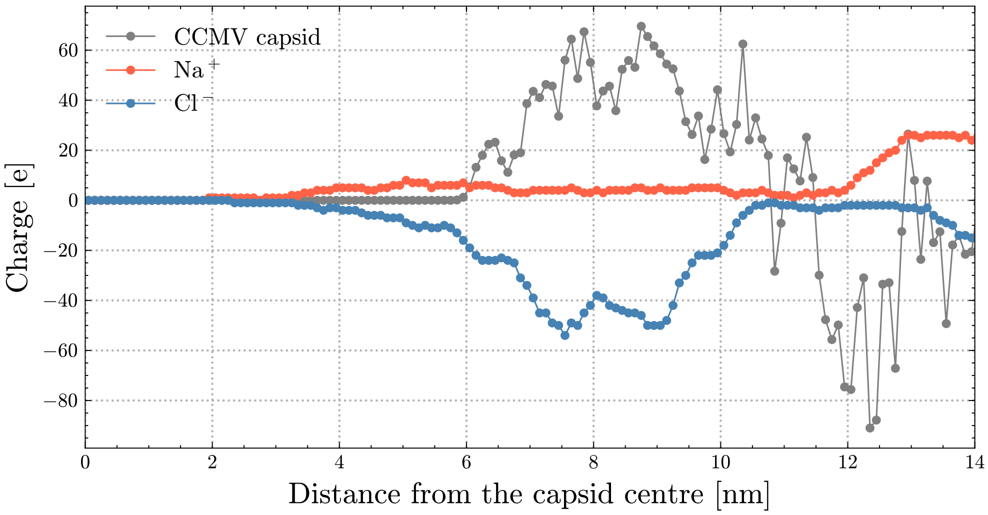

Supplementary Figure S19: Profiles of the radial charge distribution associated with the CCMV capsid and the ionic shells thereof, from the last frame of the MD minimization/thermalization protocol described in Section IV B 2. Contributions from each species are integrated over 1 Å-thick spherical shells, along the radial distance from the core of the capsid.

The calculation requires that the charge distribution be firstly discretized along the radial distance as $\rho(r) \approx \rho[r_i] \equiv \rho_i$, with i denoting a binning interval $I_i = \left[r_i - \frac{\delta R}{2}, r_i + \frac{\delta R}{2} \right]$ of thickness $\delta R = 1$ Å.

By assuming ρ_i to be spherically-symmetric, we define the charge density of the i-th shell as:

$$\rho_i = \frac{Q_i}{\frac{4}{3}\pi \left(\left(r_i + \frac{\delta R}{2} \right)^3 - \left(r_i - \frac{\delta R}{2} \right)^3 \right)} \equiv \frac{Q_i}{\frac{4}{3}\pi \delta R \left(3r_i^2 + \frac{(\delta R)^2}{4} \right)}, \tag{2}$$

with Q_i the total (net) charge from the contributions of all species within interval I_i.

Within the approximation of spherical symmetry, the radial electric field (i.e. $\mathbf{E}(\mathbf{r}) = E(r)\hat{r}$) originated by a charged shell of volume $\mathcal{V}_i$ and total charge $Q_i = \rho_i \mathcal{V}_i$ is straightforwardly derived *via* Gauss' theorem:

$$E_i(r) = \begin{cases} 0, & \text{if } r < r_i \\ \frac{Q_i}{4\pi\epsilon_0 r^2}, & \text{if } r \geq r_i. \end{cases} \tag{3}$$

Hence, the electric potential $V(r)$:

$$V(r) - V(\infty) := -\int_r^\infty dr'\ E(r') = \begin{cases} 0, & \text{if } r < r_i \\ \frac{Q_i}{4\pi\epsilon_0 r}, & \text{if } r \geq r_i \end{cases} \tag{4}$$

with $V(\infty)$ set to zero. Each shell I_i is thus subject to the electric field enforced by all layers underneath, so that at a radial distance R_i:

$$V(R_i) - V(\infty) := -\int_{R_i}^\infty dr' \left(\sum_{j=1}^{i} E_j(r') \right) \tag{5}$$

thereby obtaining a discretized version of $V_{cap}(r)$, denoted $V_{cap}[r]$. To convert this mean-field potential into an electric potential energy $U_{cap}[r] = qV[r]$, we employed a probe charge $q = q_{nuc}$ corresponding to the effective charge of the single nucleotide adopted in the oxRNA model. A Gaussian-filtered version of the electric potential energy $U_{cap}[r]$, denoted $\tilde{U}_{cap}[r]$, was fitted by a polynomial curve - as described in the main text: This expression ($U_{cap}(r)$) was lastly implemented within the oxDNA code.

C. End-to-end distance of the RNA2 fragment within the capsid-like external field

Figure **S20** shows the evolution of the end-to-end distance of RNA2, in the encapsidated MD scenarios at $T = 293$ K and $I = 0.5$ M with no harmonic bias enforced on the RNA termini. To recall, these trajectories were started from the structures obtained in the last frame of each squeezing replica at $T = 293$ K and $I = 0.5$ M, where the RNA2 termini had been harmonically constrained.

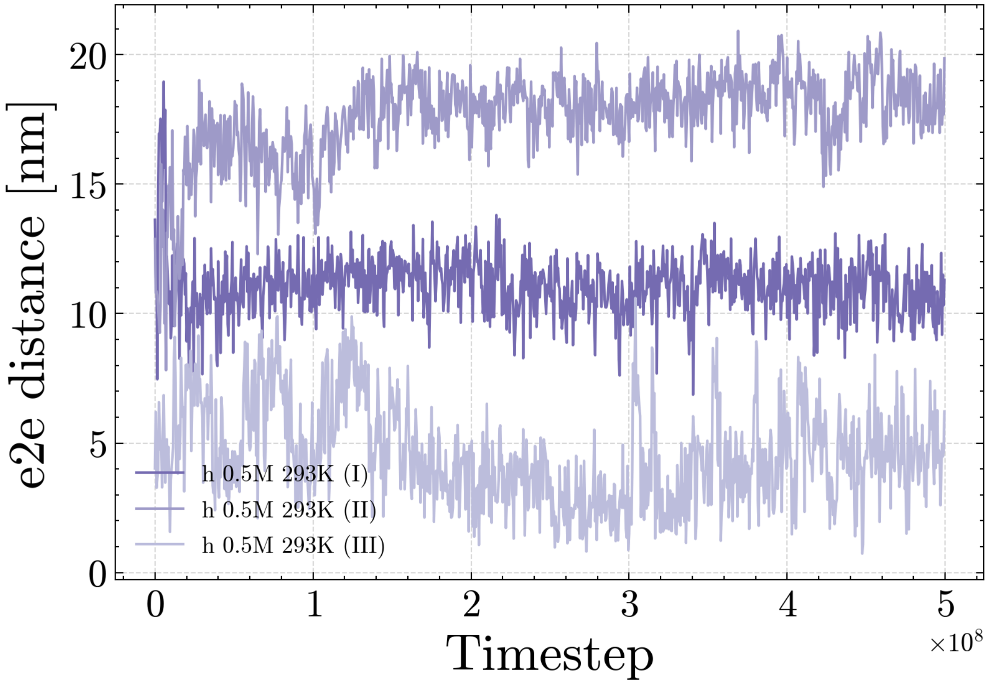

Supplementary Figure S20: Time evolution of end-to-end (e2e) distance from the encapsidated MD replicas at $T = 293$ K and $I = 0.5$ M with unconstrained RNA termini.

D. Fitting parameters

1. *Analytical approach*

Here is a list of the parameters derived from the fitting procedure of the analytical solution reported by Šiber and Podgornik [2] (Equation **2** of the main article):

- $\alpha = -4.3\ k_B T$
- $\lambda = 3.448\ \lambda_{ox}$ the Debye length,
- $\epsilon = 1 \times 10^{-4}\ k_B T$
- $\sigma = 1.9\ \lambda_{ox}$
- $R_\delta := R_0 + \delta = 15.5\ \lambda_{ox}$, to avoid divergences of the forces acting on particles close to the surface in R_0.

2. *Structure-based approach*

Here is a list of the parameters derived from the fitting procedure of the discredited potential energy $U_{cap}(r)$, obtained from the atomistic structures of the CCMV capsid as described in Section IV B 3 (Equation **3** of the main article).

- $R_0 = 14.8\ \lambda_{ox}$
- $A = -7.77913022 \times 10^{-2}\ k_B T/\lambda_{ox}$
- $c_1 = 4.59994536\ \lambda_{ox}^{-1}$
- $c_2 = -1.08939804 \times 10^{1}\ \lambda_{ox}^{-2}$
- $c_3 = 7.68423122\ \lambda_{ox}^{-3}$
- $c_4 = -2.79192908\ \lambda_{ox}^{-4}$
- $c_5 = 6.06976204 \times 10^{-1}\ \lambda_{ox}^{-5}$
- $c_6 = -8.29137297 \times 10^{-2}\ \lambda_{ox}^{-6}$
- $c_7 = 7.15232653e \times 10^{-3}\ \lambda_{ox}^{-7}$
- $c_8 = -3.77388959 \times 10^{-4}\ \lambda_{ox}^{-8}$
- $c_9 = 1.11053305 \times 10^{-5}\ \lambda_{ox}^{-9}$
- $c_{10} = -1.39522295 \times 10^{-7}\ \lambda_{ox}^{-10}$

E. Chord diagram depiction of the stable hydrogen-bonding contacts

1. Chord diagrams associated with each independent replica in the structure-based approach (T=310 K, different salt concentration and unconstrained ends)

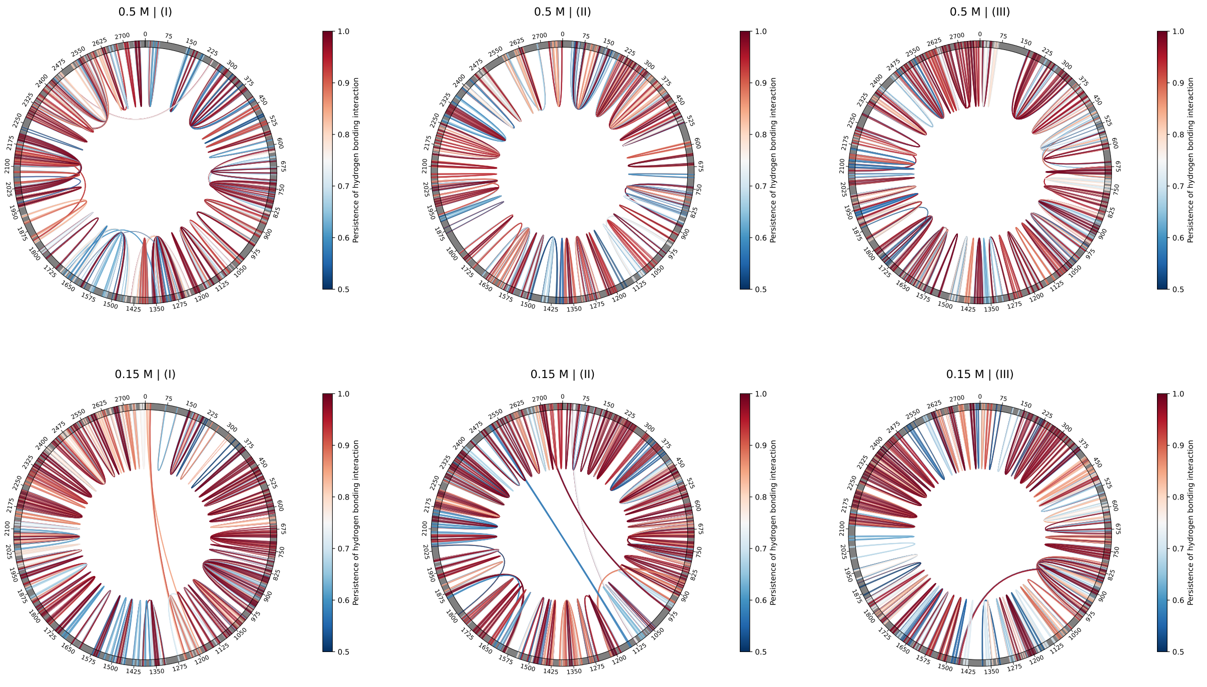

Supplementary Figure S21: Chord diagram depiction of the stable hydrogen-bonding contacts, i.e., conserved in over 50% of the trajectory frames, within the independent MD replicas. The data shown here refer to the structure-based approach.

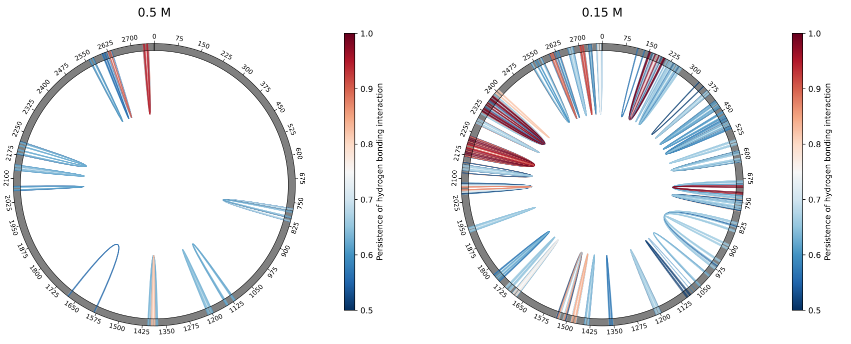

Supplementary Figure S22: Chord diagram depiction of the stable hydrogen-bonding contacts (i.e., conserved in over 50% of the total trajectory frames per salt concentration), from the structure-based approach at (left) 0.5 M and (right) 0.15 M

2. Chord diagrams associated with each independent replica in the structure-based approach (T=293 K and I=0.5 M)

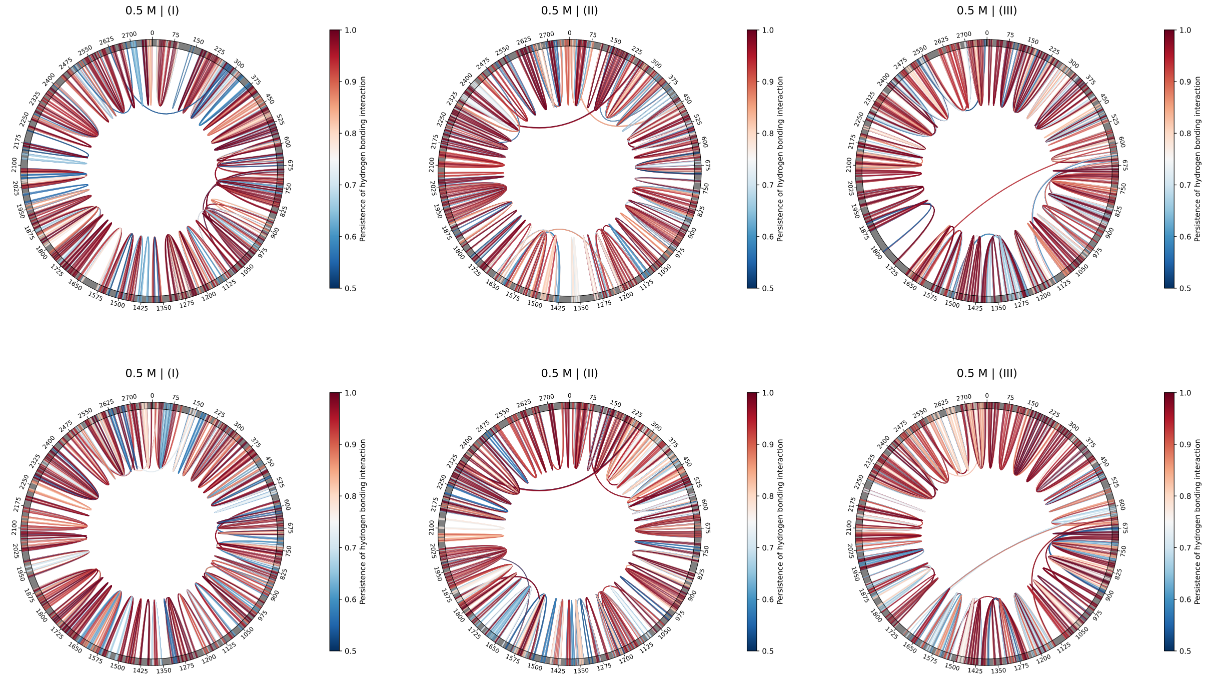

Supplementary Figure S23: Chord diagrams of the stable hydrogen-bonding contacts, i.e., conserved in over 50% of the trajectory frames within each MD replica. The top row shows the results from the encapsidated MD scenarios where the RNA2 termini were harmonically constrained, whereas the bottom row refers to the encapsidated MD scenarios with unconstrained RNA termini.

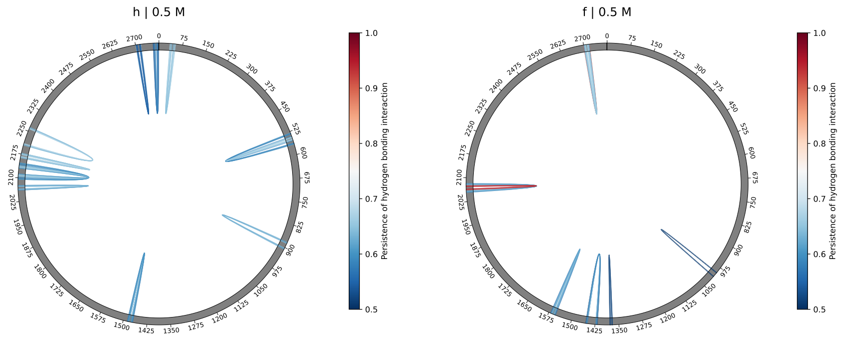

Supplementary Figure S24: Chord diagram depicting the stable hydrogen-bonding contacts, i.e., conserved in over 50% of all trajectory frames from (**left**) the encapsidated MD scenarios with harmonically-constrained RNA2 termini (labeled as **h**), and (**right**) the encapsidated MD scenarios with no constraint over the RNA2 termini (labeled as **f**).

F. Number of hydrogen-bonding interactions and density of nucleotides of RNA2 within a capsid-like electrostatic environment

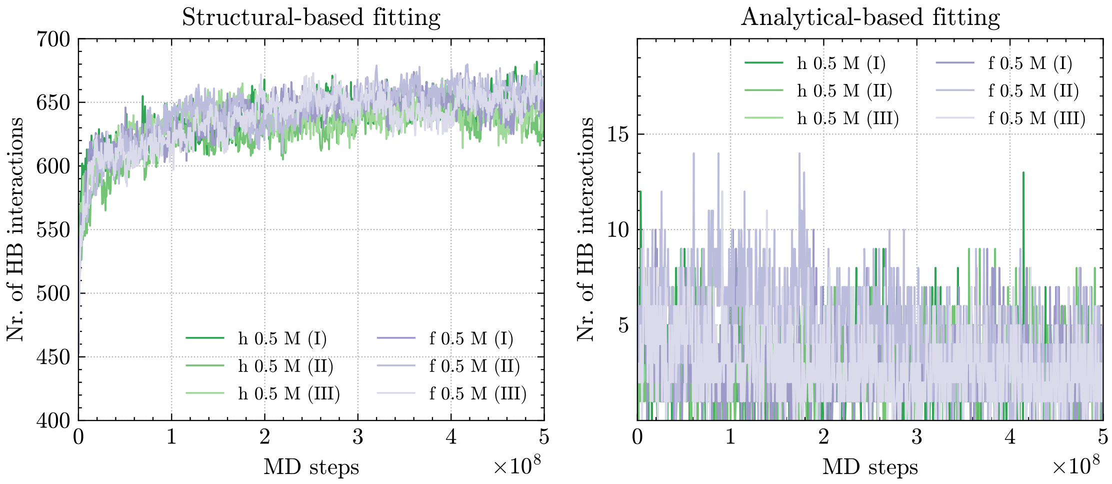

Supplementary Figure S25: The total amount of hydrogen-bonding interactions from the encapsidated MD trajectories of RNA2 at 293 K, obtained by adopting either (**left**) a structure-based ($U_{cap}(r)$), or (**right**) an analytical approach ($U_{yuk}(r)$). Simulations were carried out both with (label **h**) and without (label **f**) harmonic constraints on the RNA2 termini.

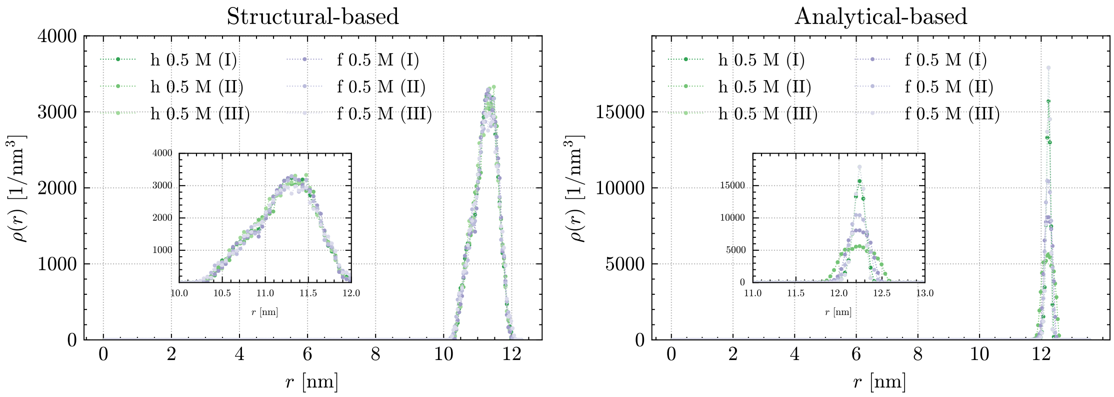

Supplementary Figure S26: Profiles of the radial nucleotide density for the MD trajectories of RNA2 at 293 K within a capsid-like electrostatic environment, derived *via* mean-field approaches relying on either (**left**) atomistic data (structure-based approach), or (**right**) theoretical calculations (analytical approach). Simulations were carried out both with (label **h**) and without (label **f**) harmonic constraints on the RNA2 termini.

V. SUMMARY OF THE SIMULATIONS PERFORMED IN THIS WORK

- I: Debye-Hückel, effective salt concentration
- T: Temperature
- Harmonic (constraint on the RNA2 termini): boolean value
- Rg: average value of the gyration radius, calculated after convergence
- nHB: number of base pairings, calculated after convergence
- PKs: boolean value reporting whether (at least) one pseudoknot is consistenty found in over 50% of the frames of at least one MD replica

Simulation Scenario	No. of Replicas	No. of steps	I	T	Harmonic	Rg	nHB	PKs
Freely-folding MD	3	10^{10}	0.15M	310K	no	$\sim$ 35 nm	$\sim$ 475	no
Freely-folding MD	3	10^{10}	0.50M	310K	no	$\sim$ 17.5 nm	$\sim$ 650	no
Freely-folding MD	3	10^{10}	0.50M	293K	no	$\sim$ 17 nm	$\sim$ 750	N/A
Freely-folding MD	3	10^{10}	0.50M	293K	yes	$\sim$ 13 nm	$\sim$ 750	yes
Freely-folding Annealing	1	10^{10}	0.15M		no	$\sim$ 30 nm	$\sim$ 600	no
Freely-folding Annealing	1	10^{10}	0.50M		no	$\sim$ 20 nm	$\sim$ 800	no
Squeezing MD	3	10^{8}-10^{9}	0.15M	310K	no	decreasing	$\sim 400 \rightarrow 700$	N/A
Squeezing MD	3	10^{8}-10^{9}	0.50M	310K	no	decreasing	550-650	N/A
Squeezing MD	3	10^{8}-10^{9}	0.50M	293K	yes	decreasing	750-800	N/A
Analytical external potential MD	3	$5 \cdot 10^{8}$	0.15M	310K	no	$\sim$ 12 nm	<10	no
Analytical external potential MD	3	$5 \cdot 10^{8}$	0.50M	310K	no	$\sim$ 12 nm	<10	no
Analytical external potential MD	3	$5 \cdot 10^{8}$	0.50M	293K	no	$\sim$ 12 nm	<10	no
Analytical external potential MD	3	$5 \cdot 10^{8}$	0.50M	293K	yes	$\sim$ 12 nm	<10	no
CCMV-like external potential MD	3	$5 \cdot 10^{8}$	0.15M	310K	no	$\sim$ 11 nm	$\sim$ 650	yes
CCMV-like external potential MD	3	$5 \cdot 10^{8}$	0.50M	310K	no	$\sim$ 11 nm	$\sim$ 600	yes
CCMV-like external potential MD	3	$5 \cdot 10^{8}$	0.50M	293K	no	$\sim$ 11 nm	$\sim$ 650	yes
CCMV-like external potential MD	3	$5 \cdot 10^{8}$	0.50M	293K	yes	$\sim$ 11 nm	$\sim$ 650	yes

[1] R. Lorenz, S. H. Bernhart, C. Höner zu Siederdissen, H. Tafer, C. Flamm, P. F. Stadler, and I. L. Hofacker, Viennarna package 2.0, Algorithms for molecular biology **6**, 1 (2011).

[2] A. Šiber and R. Podgornik, Role of electrostatic interactions in the assembly of empty spherical viral capsids, Physical Review E—Statistical, Nonlinear, and Soft Matter Physics **76**, 061906 (2007).

[3] J. A. Speir, S. Munshi, G. Wang, T. S. Baker, and J. E. Johnson, Structures of the native and swollen forms of cowpea chlorotic mottle virus determined by x-ray crystallography and cryo-electron microscopy, Structure **3**, 63 (1995).

[4] E. F. Pettersen, T. D. Goddard, C. C. Huang, G. S. Couch, D. M. Greenblatt, E. C. Meng, and T. E. Ferrin, Ucsf chimera—a visualization system for exploratory research and analysis, Journal of computational chemistry **25**, 1605 (2004).

[5] A. Fiser and A. Šali, Modeller: generation and refinement of homology-based protein structure models, in *Methods in enzymology*, Vol. 374 (Elsevier, 2003) pp. 461–491.

[6] All minimization and equilibration steps hereby described were carried out *via* the 2018 version of the Gromacs toolkit [9].

[7] J. Huang, S. Rauscher, G. Nawrocki, T. Ran, M. Feig, B. L. De Groot, H. Grubmüller, and A. D. MacKerell Jr, Charmm36m: an improved force field for folded and intrinsically disordered proteins, Nature methods **14**, 71 (2017).

[8] P. L. Freddolino, A. S. Arkhipov, S. B. Larson, A. McPherson, and K. Schulten, Molecular dynamics simulations of the complete satellite tobacco mosaic virus, Structure **14**, 437 (2006).

[9] M. J. Abraham, T. Murtola, R. Schulz, S. Páll, J. C. Smith, B. Hess, and E. Lindahl, Gromacs: High performance molecular simulations through multi-level parallelism from laptops to supercomputers, SoftwareX **1**, 19 (2015).
